# Biomaterials’ enhancement of immunotherapy for breast cancer by targeting functional cells in the tumor micro-environment

**DOI:** 10.3389/fimmu.2024.1492323

**Published:** 2024-11-12

**Authors:** J. Paul Santerre, Yangyang Yang, Ziwei Du, Wenshuang Wang, Xiaoqing Zhang

**Affiliations:** ^1^ The School of Basic Medicine, Binzhou Medical University, Yantai, Shandong, China; ^2^ Translational Biology and Engineering Program, Ted Rogers Centre for Heart Research, Toronto, ON, Canada; ^3^ Department of Gynecology, the Affiliated Yantai Yuhuangding Hospital of Qingdao University, Yantai, Shandong, China

**Keywords:** breast cancer, immunotherapy, biomaterials, tumor micro-environment, nanoparticles, immune cells

## Abstract

Immunotherapy for breast cancer is now being considered clinically, and more recently, the number of investigations aimed specifically at nano-biomaterials-assisted immunotherapy for breast cancer treatment is growing. Alterations of the breast cancer micro-environment can play a critical role in anti-tumor immunity and cancer development, progression and metastasis. The improvement and rearrangement of tumor micro-environment (TME) may enhance the permeability of anti-tumor drugs. Therefore, targeting the TME is also an ideal and promising option during the selection of effective nano-biomaterial-based immuno-therapeutic strategies excepted for targeting intrinsic resistant mechanisms of the breast tumor. Although nano-biomaterials designed to specifically release loaded anti-tumor drugs in response to tumor hypoxia and low pH conditions have shown promises and the diversity of the TME components also supports a broad targeting potential for anti-tumor drug designs, yet the applications of nano-biomaterials for targeting immunosuppressive cells/immune cells in the TME for improving the breast cancer treating outcomes, have scarcely been addressed in a scientific review. This review provides a thorough discussion for the application of the different forms of nano-biomaterials, as carrier vehicles for breast cancer immunotherapy, targeting specific types of immune cells in the breast tumor microenvironment. In parallel, the paper provides a critical analysis of current advances/challenges with leading nano-biomaterial-mediated breast cancer immunotherapeutic strategies. The current review is timely and important to the cancer research field and will provide a critical tool for nano-biomaterial design and research groups pushing the clinical translation of new nano-biomaterial-based immuno-strategies targeting breast cancer TME, to further open new avenues for the understanding, prevention, diagnosis and treatment of breast cancer, as well as other cancer types.

## 1 Introduction of breast cancer and immunotherapy

Breast cancer, which is referred to as a complex and heterogeneous disease, is one of the most common malignant tumors in women with a high mortality rate (1, 2). Both genetic and external risk factors can contribute to its incidence (1, 2). The main risk factors for breast cancer include sex, age, genetic factors, hormone therapies, lifestyles and dietary habits etc. (3). The conventional treatment options for breast cancer include surgery, chemotherapy and radiotherapy in clinical practices (4, 5). However, in some instances, recurrence is inevitable due to the undetected or residual breast cancer tissues (4, 5). In addition, therapeutic resistance is also a significant obstacle for the effective treatment of breast cancer, due in part to the distinctive molecular profiles and biological characteristics of the different breast cancer subtypes (6).

Generally, based on the presence or absence of specific receptors on the cell surface, breast cancer can be classified into three main subtypes, estrogen receptor (ER) and/or progesterone receptor (PR) positive (Luminal A/B, based on Ki-67 expression), human epidermal growth factor receptor 2 (HER2) positive, and triple negative breast cancer (TNBC) (7). Therefore, different therapeutic approaches have been introduced to treat the distinctive breast cancer subtypes to prolong the progression-free survival and overall survival of the patients. Normally, endocrine therapy is used for breast cancer types that express hormone receptors. In addition, chemotherapy and inhibitor treatments, targeting the cell cycle or specific receptor-related signaling pathways, are also common for patients with hormone receptor-positive breast cancer (7). Monoclonal antibodies and chemotherapy are commonly used in HER2 positive breast cancer patients (8). However, TNBC, which accounts for 10%-20% of all breast cancer cases, has been considered to be the most aggressive subtype and the most challenging one to treat because of its deficiency of ER, PR and HER2 (9). Since TNBC cells lack the expression of druggable cell-surface target receptors, specific targeted therapy for TNBC cannot be proposed, instead, TNBC patients are often treated with neoplastic drugs and chemotherapies (10).

The immune system plays a vital role during the progression and regression of breast cancer (11). While the traditional radiotherapy and chemotherapy focuses on directly killing the tumor cells, immunotherapy works by activating the patients’ own immune systems to recognize and combat breast cancer cells (including the abscopal metastatic tumors) (12, 13). In addition, immunotherapy is also endowed with the ability to impede tumor metastasis and recurrence (12, 13). Recently, the application of immunotherapy exhibited the potential for improved clinical outcomes in cancer treatment (14, 15). As the most invasive type of breast cancer, TNBC was perceived as the most immunogenic type due to its high genome instability, increased mutation rates, enhanced tumor antigen production and frequent lymphocytic infiltration (4, 16). Programmed death-ligand 1 (PD-L1), which is a negative regulator in the immune system, has been shown to be highly expressed in TNBC (17, 18), indicating the feasibility of immunotherapy for TNBC.

The current approaches for cancer immunotherapy include adoptive cell therapy (e.g., tumor-infiltrating lymphocyte (TIL), chimeric antigen receptor (CAR) T-cell, T-cell receptor-engineered (TCR) T-cell, chimeric antigen receptor-natural killer cell (CAR-NK) therapies), and immune checkpoint blockade and cancer vaccine therapies (7, 12). Specifically, the current immunotherapies utilized in breast cancer treatment comprise of tumor-targeting antibodies, adoptive T cell therapy, cancer vaccines and immune checkpoint blockade (4). Among those different strategies, monoclonal antibodies, such as trastuzumab and pertuzumab targeting HER2, have shown efficacy, and been widely applied clinically (19). In addition, cancer vaccines including DNA and peptide vaccines have been reported to exhibit their efficacy by activating the immune system in breast cancer therapeutic regimens (20–22). Although the latter breast cancer immunotherapies have shown promise, limitations and challenges have also been identified. For example, the frequent and unpredictable mutations in breast tumor cells may decrease the drug response and targeting efficiency of monoclonal antibodies (4). Moreover, the lack of appropriate tumor antigens and the immunosuppressive tumor microenvironment can limit the application of tumor vaccines (4). To enhance the therapeutic efficacy of breast cancer treatments, the combination of multiple immunotherapies may offer an alternative option.

Immune checkpoint inhibition, such as the monotherapy targeting the negative immune checkpoint molecules, programmed cell death protein 1 (PD-1) or cytotoxic T-lymphocyte-associated protein 4 (CTLA-4), have great efficacy in the treatment of solid tumors (23). CTLA-4 is a specific target in antibody-based immunotherapy due to its competition with CD28 (on T cells) for the binding with CD80/B7-1 and CD86/B7-2 on the antigen-presenting cells (APCs), inhibiting the T-cell function (24–27). Additionally, the PD-1/PD-L1 axis also plays an inhibitory role in T-cells’ activation. The antibodies targeting this axis could enhance T-cells’ anti-tumor activity (28). In a recent phase II clinical trial for breast cancer, simultaneous inhibition of PD-1 and CTLA-4 by durvalumab and tremelimumab respectively, exhibited clinical beneficial effects in 71% of the enrolled TNBC patients (28). Further, the combination of immune checkpoint inhibition and conventional cancer therapeutic approaches provide another option to boost the treatment effects. For instance, the combination of atezolizumab (anti-PD-L1 antibody) and nab-paclitaxel (chemotherapeutic agent) demonstrated better effects in treating metastatic TNBC vs. antibody treatment or chemotherapy alone in clinical trials (23). This combinatorial treatment has also recently been approved by the U.S. Food and Drug Administration (FDA) for locally advanced or metastatic PD-L1^+^ TNBC (23). Similarly, pembrolizumab (anti-PD-1 antibody) plus chemotherapy was tested for treating locally recurrent, unresectable, or metastatic TNBC. The latter was also approved by the FDA (23). Further, the combined application of trastuzumab (monoclonal antibody targeting HER2) and HER2 vaccine showed better effects in treating HER2^+^ breast cancer patients vs. trastuzumab or HER2 vaccine treatment alone (29). However, it has been noted that the systemic long-term use of monoclonal antibodies targeting immune checkpoint inhibition can cause immune-related adverse effects in a large number of patients, reducing the immune therapy efficacy (30). Consequently, the adequate delivery and the stable release of the drugs are key factors to consider for efficiently activating the anti-tumor immune responses in the breast cancer microenvironment.

Currently, there are review articles that have discussed the drug-delivery technologies designed to improve the effectiveness of *in situ* vaccine-based anti-breast cancer therapies (31). In addition, review papers have also covered the application of nano-biomaterials that could respond to physiological stimuli within breast tissue, and how they can be loaded with both drugs and gene products (e.g., siRNAs, RNAi and noncoding RNAs) and have controlled release at the tumor target site to improve the conventional strategies for treating breast cancer (32). However, the existing review papers have not yet discussed the important role that breast cancer tumor-micro-environment (TME), which contains both breast tissue cells and immune cells that reside within the local breast tissue niche (e.g., macrophages, cancer-associated fibroblasts, myeloid-derived suppressor cells, dendritic cells, natural killer cells and T lymphocytes) and the growth factors/cytokines produced/released, could have played in regulating breast cancer progression and metastasis. The lack of appreciation of the importance of targeting breast cancer TME and the specific immune cell types within the different subtypes of breast cancer could significantly hinder the effectiveness of current anti-breast cancer therapeutics. As a result, this manuscript thoroughly discusses the mechanisms underlying how breast cancer TME, as well as the different immune cells can modulate breast tumor progression/metastasis. Additionally, some of the most advanced progress regarding the use of innovative biomaterials for targeting the immunosuppressive cells and immune cells in the TME, to improve the breast cancer treating outcomes are thoroughly examined. Further, the manuscript critically evaluates the limitations of the current biomaterial-based immunotherapy targeting breast cancer and the potential future research directions of the field have been pointed out.

This current paper would significantly enhance the field’s understanding of how breast cancer TME (tissue cells, immune cells, growth factors/cytokines/chemokines) could lead to the development of the various breast cancer subtypes and modulate the breast cancer progression characteristics, as well as provide great insights into innovative biomaterial and drug designs to precisely target specific cell types to boost immunotherapy efficiency.

## 2 The tumor micro-environment (TME)

TME is referred to as the special ecosystem that surrounds a tumor inside the body, which includes the resident and recruited host cells (such as cancer-associated fibroblasts (CAFs) and immune cells), cell products (such as pro-inflammatory cytokines and chemokines), growth factors, extracellular matrix (ECM), stromal cells, blood vessels, lymphatic vessels and other cells or biomolecules closely associated with the tumor cells (33–35). The complex cellular and non-cellular components in TME not only participate in the initiation, progression and metastasis of cancers, but also respond to the specific therapeutic regimen (36). For the different cell types within the TME, inflammatory cells and fibroblasts have been found to participate in key processes such as angiogenesis and ECM remodeling, resulting in the uncontrolled growth and metastasis of tumor cells (37, 38). Stromal cells, immune cells and non-stromal factors, could promote the resistant phenotype and affect the efficacy of chemotherapy during chemotherapy for breast cancer (35). Alterations of the breast cancer micro-environment can play a critical role in antitumor immunity and cancer development, progression and metastasis (39). And the improvement and rearrangement of TME may enhance the permeability of anti-tumor drugs (40). Therefore, targeting the TME is also an ideal and promising option during the selection of effective therapeutic strategies excepted for targeting intrinsic resistant mechanisms of the breast tumor (41).

In recent years, nanotechnology has opened up a new avenue for breast cancer treatment (42). Specifically, biomaterials-based nanoparticles (NPs) that exhibit both drug delivery and stimulative functions hold great promise for breast cancer therapeutics and there are some nano-platforms that have been approved by the FDA (6, 43). Many NP-based cancer medications have been evaluated or applied in clinical trials, for example, NP albumin-bound-paclitaxel is one of the most widely used nano-medication in cancer treatment (44). Some properties of NPs such as small sizes, large surface area, high surface-volume ratio, high surface reactivity, unique physicochemical properties, enhanced permeability and retention (EPR) effects and superior reactivity over their bulk counterparts enable their application potential in the early diagnosis and improved treatment of breast cancer (6, 43). Since NPs could increase tumor immunogenicity by generating free radicals, NP-based therapeutics delivery systems can enhance the immunotherapy efficacy of breast cancer (45, 46). NPs can also be designed to have good biocompatibility, minimal cytotoxicity, targeted accumulation in solid tumors and enhanced drug delivery efficiency (13, 47–49). Specifically, the anti-breast cancer drug and the assisted NP delivery system could be designed based on the tumor tissue pathological characteristics (50, 51). The distinct physical, chemical and biological properties between the normal and tumor tissues, such as pH of the tissue micro-environment, oxidation-reduction (redox) state, temperature, oxygen level and the differently expressed genes and proteins, endow the possibility of the targeted delivery of biomaterial-carried anti-tumor drugs (50, 51).

Biomaterials, designed to specifically release loaded anti-tumor drugs in response to tumor hypoxia and low pH conditions, have emerged in recent years (52). What is more, the diversity of TME components also supports a broad targeting potential for anti-tumor drug designs (53, 54). Encouragingly, TME-targeting therapeutic strategies, enabled with specific biomaterials, could potentially exhibit advantages such as precise targeted delivery for anti-tumor drugs or biomacromolecules, and reduced risks of side effects from the treatments, paving a new way for more effective cancer treatments when compared to the traditional chemotherapy or radiotherapy. Consequently, biomaterial-assisted immunotherapies have been considered as an alternative strategy to effectively treat cancer patients and need to be extensively explored. Some current progress regarding the use biomaterials for targeting immunosuppressive cells and immune cells in the TME, for improving the breast cancer treating outcomes are discussed in Sections 3&4.

## 3 Biomaterials targeting immunosuppressive cells in breast cancer TME

The solid TME is often very complex due to the presence of immunosuppressive cells and factors (55, 56). The immunosuppressive cells in TME, such as tumor-associated macrophage (TAMs), CAFs, regulatory T cells (Tregs), myeloid-derived suppressor cells (MDSCs) could influence the immunotherapy effects in the tumors (57). The application of biomaterials toward specifically targeting those immunosuppressive cells or factors in the TME could be a powerful strategy to augment the breast cancer treatment outcomes. For example, biomaterials can be designed to be effective cargo carriers to deliver specific drugs to enhance immunogenicity, improve antigen presentation or T cell infiltration, to eventually contribute to the remodeling of the tumor TME. In this section, how biomaterials have been designed to target the different immunosuppressive cells of the breast cancer TME, toward boosting the therapeutic effects, are discussed in greater detail.

### 3.1 M2-like tumor-associated macrophages (TAMs)

Macrophages are distributed in all tissues across the human body and play a vital role in innate immunity (58, 59). Macrophages originate from monocytic precursors in blood and differentiate in the presence of cytokines and growth factors in tissues (58, 59). Macrophage abnormalities can result in disease progression such as tissue fibrosis, diabetes, and different cancers (60). In tumors, the TME influences the recruitment and polarization of macrophages and determines the pro-tumorigenic outcomes (58, 60, 61). During breast tumor progression, macrophages can cause inflammatory responses, promote tumor cell growth, angiogenesis, migration and invasion of breast cancer cells, ultimately progressing to malignancy (58, 60, 61).

#### 3.1.1 M2-like TAMs in breast cancer TME

Macrophages can change their phenotypes in response to tumor microenvironmental cues and become TAMs (61). As a major cellular component of TME, TAMs have recently been found to be present in multiple malignant tumor types (60, 62, 63). Generally speaking, M1 macrophages play an anti-tumor role, while the TAMs are more of a M2 polarization phenotype and have tumor-promoting properties (64). Therefore, M2 TAMs were regarded as an attractive therapeutic target for cancer immunotherapy, due to their pivotal role in cancer progression phases including angiogenesis, immune suppression, hypoxia induction, invasion, and metastasis (58, 65, 66).

Tissues isolated from TNBC patients have demonstrated a high infiltration of M2-like TAMs, and those patients typically have poor prognosis (35, 66). Considering the relationship between TAMs’ abundance and breast cancer patients’ prognosis, removal of the M2-like TAMs, restoration of TAMs’ immunostimulatory phenotype or inhibition of TAMs’ tumor-promoting functions might be effective breast tumor targeting therapies (6, 60). However, it should be noted that the extensive distribution of macrophages and the multifaceted roles that those cells play in different tissues across the body increases the difficulty for the effective delivery of therapeutic drugs specifically to the tumor-promoting M2-like TAMs (64).

#### 3.1.2 Biomaterial-assisted immunotherapeutic approaches targeting M2-like TAMs

Some biomaterial-assisted immunotherapeutic approaches have been developed to specifically block the survival of M2-like TAMs, switch the cells’ phenotypes from M2- to M1-like, or directly kill the M2-like TAMs, to achieve better breast tumor control outcomes. More specifically, the switch of TAMs’ polarization states (M2 to M1) can be achieved by modulating the signaling pathways associated with macrophage activation (67, 68).

The binding of macrophage colony stimulating factor (MCSF) secreted by cancer cells with colony stimulating factor 1 receptor (CSF1-R, a class III tyrosine receptor) expressed on macrophages can promote the M2 polarization of TAMs (67). Ramesh et al. reported a stable supramolecular nano-assembly formed by CSF1-R, Src homology region 2 (SH2) domain-phosphatase (SHP2) inhibitors and co-lipid based phospholipid-polymer conjugates (1, 2-distearoyl-sn-glycero-3-phosphoethanolamine-poly(ethylene-glycol) (DSPE-PEG) and phosphatidyl choline) (67). The self-assembled dual-inhibitor-loaded NPs (DNPs) could target M2 TAMs to inhibit the MCSF-CSF1-R and CD47-signal regulatory protein α (SIRPα) pathways simultaneously, resulting in their repolarization to anti-tumorigenic M1 phenotypes, and enhancing their phagocytic function (67). What is more, DNPs showed high anti-tumor efficacy, minimal normal tissue toxicity in breast cancer mouse models, indicating their future promise for applications in breast cancer immunotherapy (67).

**Figure 1:**
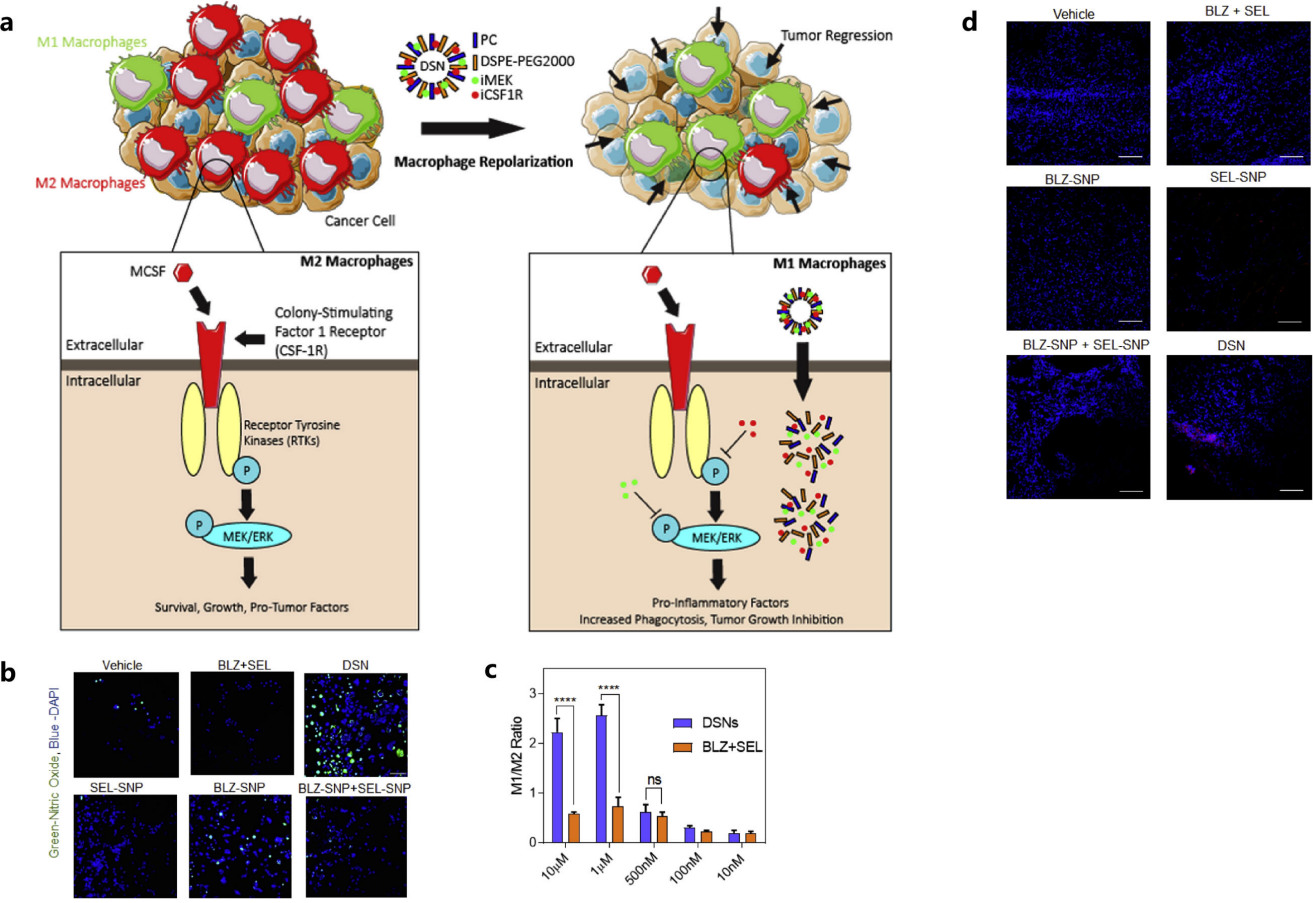
Mechanism of the action of dual-kinase inhibitor-loaded supramolecular nanoparticles (DSN) targeting CSF1R and MEK in macrophages **(A)**, confocal images **(B)** and flow cytometry plots **(C)** showed the repolarization of M2 to M1 phenotype of the macrophages, tumor cell deaths in DSN treatment by confocal analysis **(D)**. Reprinted with permission from Ramesh A, Brouillard A, Kumar S, et al. (68). Copyright 2019, Elsevier. ns means not significant, ****p<0.0001 (one-way ANOVA).

Additionally, the mitogen-activated protein kinase (MAPK) signaling pathway, a downstream signaling pathway of CSF1R, also plays a vital role in modulating the proliferation, survival and functional activity of M2 macrophages (68). Ramesh et al. also achieved the concurrent inhibition of CSF-1R and MAPK signaling pathways by synthesizing NPs using supramolecular self-assembly, to further improve the anti-breast cancer effects of macrophage-based immunotherapy (68). The NPs were synthesized in the presence of phosphatidyl choline and DSPE-PEG2000-amine with amphiphilic dual-kinase inhibitors targeting CSF1R and MEK (68). These supramolecular NPs could accumulate in TAMs at a high concentration and reprogrammed the polarization of immunosuppressive M2-like macrophages to M1-like phenotype in a highly aggressive 4T1 breast cancer model (**Figure 1**) (68). The design of those NPs carrying inhibitors, targeting specific signaling pathways that can modulate M2-like TAMs’ phenotypic switches, showed effectiveness in remodeling of the immunosuppressive breast cancer TME (68).

The intratumoral injection of stimulators of interferon gene (STING) agonists could have some benefits for inhibiting breast cancer progression, however, the uneven diffusion and distribution of agonists limited the treatment efficiency (69). It can be seen that liposomal NPs can improve the diffusion and distribution of loaded drugs in tumors for better treating outcomes. Since cyclic guanosine monophosphate–adenosine monophosphate (cyclic GMP-AMP, cGAMP) is an intracellular second messenger that can activate the innate immune STING pathway, Cheng et al. designed liposomal NPs to deliver cGAMP for STING receptors in TNBC (70). Liposomal NP-delivered cGAMP reprogrammed the M2-like macrophages into M1-like macrophages in the TME, which was also accompanied with increased major histocompatibility complex (MHC) expression and CD4^+^ and CD8^+^ T cell infiltration for enhanced anti-breast tumor immunity (70). This NP-assisted delivery strategy exhibited more effectively activated STING than direct loading of cGAMP, without NP assistance in the treatment of PD-L1 insensitive TNBC.

**Figure 2:**
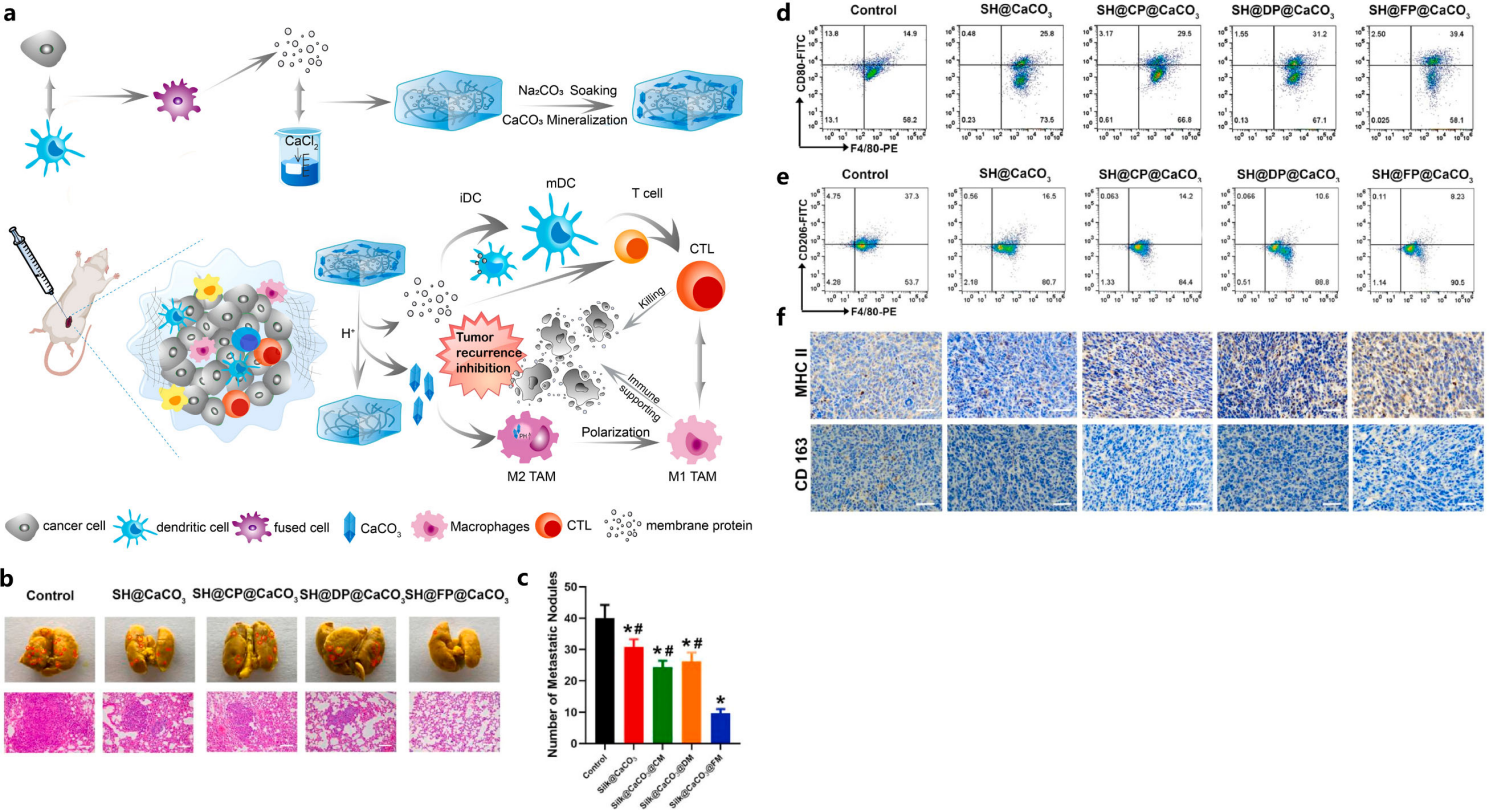
Illustration of FP-containing biomineralized hydrogel vaccine (SH@FP@CaCO_3_) that inhibited tumor recurrence **(A)**, effects of SH@FP@CaCO_3_ on tumor metastasis **(B, C)**, reduction of M2-type macrophages with SH@FP@CaCO_3_ treatment confirmed by flow cytometry **(D, E)** and IHC **(F)**. Reprinted with permission from Huo W, Yang X, Wang B, et al. (71). Copyright 2022, Elsevier. *p<0.05 vs. control, ^#^p<0.05 vs. SH@FP@CaCO_3_.

In addition to the liposomal NPs, a hydrogel was also developed to improve the TME. Huo et al. reported the development of a CaCO_3_ biomineralized silk fibroin hydrogel-based dendritic cell vaccine, to further improve the immunogenicity and remodel the immunosuppressive TME for efficient TNBC immunotherapy (71). This silk fibroin-based dendritic cell vaccine was generated by fixing the 4T1 cells-DCs fusion cells’ (FPs’) membrane proteins into biomineralized CaCO_3_ silk fibroin hydrogel (71). The CaCO_3_ dendritic cell vaccine presented defined tumor-associated antigens and achieved sustained membrane protein release, which enhanced the immunogenicity and promoted the maturation and activation of dendritic cells and T cells within the TME (71). Additionally, the effective incorporation of CaCO_3_ in the hydrogel vaccine, promoted the polarization of M2-like macrophages to M1-like macrophages, increased pH of TME, reversed the immune-inhibitory state of the TME, and reduced the immunosuppression of T cells (**Figure 2**) (71).

On the other hand, strategies for deleting M2-like TAMs in the TME were also explored in other cancer types. For example, M2-like TAM dual-targeting NPs (M2NPs), which were biocompatible fusion peptide-functionalized lipid NPs, were fabricated to specifically inhibit the survival of M2-like TAMs, and eventually delete them (64). Specifically, anti-CSF1-R siRNA (siCD115) was loaded on M2NPs, which structure and function were controlled by α-peptide (a scavenger receptor B type 1 (SR-B1) targeting peptide) linked with M2pep (an M2 macrophage binding peptide) (64). M2NPs efficiently delivered siCD115 into M2-like TAMs, which blocked the survival signaling pathway of M2-like TAMs and eliminated them (64). Further, M2NP-siCD115 exhibited the ability to reprogram the cytokine profile in the breast cancer TME (inhibited immunosuppressive IL-10 and transforming growth factor beta (TGF-β) production and boosted the immunostimulatory IL-12 and IFN-γ expression) and restored the IFN-γ secretion of tumor-infiltrated CD8^+^ T cells (64). Similarly, Jin et al. developed a melittin-RADA32-doxorubicin (DOX) hydrogel (MRD hydrogel) that exhibited interweaving nanofiber structures and provided good biocompatibility to actively regulate TME (72). The MRD hydrogel offered a melittin and DOX-based direct effect to specifically delete M2-like TAMs, activate dendritic cells of the draining lymph nodes, and produce more active cytotoxic T cells within the immunosuppressive TME (72). Due to its effects on tumor inhibition or ablation, the MRD hydrogel has shown great potential to be applied as a powerful immunotherapy in the prevention of local tumor recurrences after surgical resections in breast cancer.

Moreover, except the reverse of immunosuppressive TME, Song et al. reported the significance of adjusting the lymph nodes’ immunosuppressive microenvironment for effective anti-breast cancer immunotherapy (73). The researchers designed albumin NP, Nano-PI, containing phosphatidylinositol 3-kinase γ (PI3Kγ) inhibitors IPI-549 and paclitaxel (PTX) (73). Nano-PI achieved immunomodulator delivery to macrophages in both tumor sites and the lymph nodes, with the combination of α-PD1. M2 to M1 macrophage repolarization occurred in the microenvironment of both the tumor tissue and the lymph nodes (73). At the same time, increased infiltration of CD4^+^ and CD8^+^ T cells, B cells, and dendritic cells, and T cell exhaustion prevention, was demonstrated. The combination of Nano-PI and α-PD1 achieved the modulation of the immune microenvironment in both tumors and lymph nodes (73). Aimed at breast cancer bone metastases, biomaterials have represented a promising approach. For instance, a multi-functional CePO_4_/chitosan (CS)/graphene oxide (GO) scaffold was formed by hydrated CePO_4_ nanorods, bioactive CS and GO NPs, which promoted the switch of M2 macrophages to M1 phenotype by releasing Ce^3+^ ions (74). As a result, this CePO_4_/CS/GO scaffold-based treating method induced the apoptosis of breast cancer cells, inhibited angiogenesis, bone metastases and promoted tissue with osteo-inductive capabilities (74).

Finally, NP according to the specific biological properties of macrophages, to enhance treating performance for lung metastasis of breast cancer, was designed. Isolated macrophage membranes were coated on emtansine liposome, conferring the biomimetic functions of macrophage. This system promoted the targeting specificity to tumor metastatic sites, and improved the delivery efficiency (43).

Overall, TAMs play a significant role in immuno-regulation and breast tumor developmental phases (75, 76). Different biomaterial-based strategies could be considered according to the specific characteristics of TAMs in a given breast cancer TME. For example, repolarizing M2 TAMs into M1 phenotypes, blocking M2 TAMs tumor-promoting activities or directly killing the M2 TAMs can be used alone or together for treating different breast cancer patients. The application of multifarious biomaterials, such as different liposomal NPs, biocompatible hydrogels (e.g., silk fibroin), graphene-modified nanorods combined with biomolecules specifically targeting M2 TAMs in breast cancer TME, holds great potential for treating breast cancer clinically in the future. The representative studies that explored the use of biomaterials targeting M2 TAMs for breast cancer treatments are summarized in **Table 1**.

**Table 1:** The use of biomaterials targeting M2-like tumor associated macrophages in breast cancer.

Biomaterial types	Effective composition of the biomaterial	Biomaterial target	Effects	References
Supramolecular self-assembly nanoparticle (liposome nanoparticle)	SHP2 inhibitors (SHP099), amphiphilic CSF1R-inhibitor	M2 macrophages	• Simultaneously inhibit CSF1R and SHP2 pathways • Repolarize M2 macrophages to M1 phenotype • Enhance phagocytic capabilities • Improve anti-cancer efficacy in aggressive 4T1 breast cancer mouse model	(67)
Supramolecular self-assembly nanoparticle (liposome nanoparticle)	Amphiphilic MEK-inhibitor and CSF1R-inhibitor	M2 macrophages	• Repolarize M2 macrophages to M1 phenotype, improve anti-tumor efficacy	(68)
Liposomal nanoparticle	cGAMP	M2 macrophages	• Repolarize M2 macrophages to M1 phenotype • Increase MHC expression and CD4^+^ and CD8^+^ T cell infiltration • Increase IFN-γ–producing T cells • Augment tumor apoptosis and prevent the formation of secondary tumors	(70)
Biomineralized CaCO_3_ silk fibroin hydrogel	4T1-dendritic fusion cells’ membrane proteins, CaCO_3_	Dendritic cells, T cells, M2 macrophages	• Promote the maturation and activation of dendritic cells and T cells within the TME • Increase the pH of TME • Promote the polarization of M2 macrophages to M1 phenotype	(71)
Albumin nanoparticle	PI3Kγ inhibitor (IPI-549), paclitaxel (PTX)	M2 macrophages	• Promote the polarization of M2 macrophages to M1 phenotype • Increase infiltration of CD4^+^ and CD8^+^ T cells, B cells and dendritic cells • Prevent T cell exhaustion	(73)
CePO_4_/CS/GO scaffold made of graphene oxide (GO) nanoparticles, hydrated CePO_4_ nanorods and bioactive chitosan (CS)	GO, CePO4, CS	M2 macrophages	• Promote the polarization of M2 macrophages to M1 phenotype • Promote blood vessel formation • Facilitate bone tissue regeneration	(74)
Macrophage membrane coating-nanoparticle	Emtansine, macrophage membrane	Cancer cells	• Inhibit cell viability • Suppress cancer metastasis	(43)

CSF1R, colony stimulating factor 1 receptor; SHP2, Src homology region 2 (SH2) domain-phosphatase; cGAMP, cyclic [G(3′,5′)pA(3′,5′)p]; MHC, major histocompatibility complex; TME, tumor microenvironment; PI3Kγ, phosphatidylinositol 3-kinase γ.

### 3.2 Cancer-associated fibroblasts (CAFs)

CAFs possess a myofibroblastic phenotype and could be found both in primary and metastatic tumors, occupying a great proportion in the tumor stromal cell population (77–79). In breast cancer, CAFs could originate from breast tissue resident fibroblasts, bone marrow-derived fibroblasts, adipocytes, mesenchymal stem cells or pericytes (80, 81). Generally speaking, CAFs can be activated by tumor cell-derived TGF-β and their activation could be sustained by more TGF-β released from the activated CAFs in the whole population (36, 41). The activated CAFs could further secrete a series of growth factors and cytokines/chemokines to modulate the tumor cells and TME, including hepatocyte growth factor (HGF), epidermal growth factor (EGF), TGF-β, interleukins (such as IL-1, IL-4, IL-6, IL-8, IL-10), chemokines (such as CXCL1, CXCL12, CXCL14, CCL2, CCL5, CCL7) (80–82).

#### 3.2.1 CAFs’ interactions with other cell types within breast cancer TME

CAFs could participate in ECM remodeling by producing ECM proteins or matrix metalloproteinases (MMPs) such as several types of collagens, fibronectin, MMP2, MMP9 (41, 80). This indicates the enabling role of CAF-mediated ECM to remodel in tumor cell migration, invasion and metastasis (41, 80). The complex interactions between CAFs and components in the tumor TME, including cancer cells, T cells, myeloid cells, and endothelial cells, has enabled their critical role in the progression of cancer, ECM remodeling, and metastasis, and has also made them an important potential targeting point for cancer immunotherapy (80).

What is more relevant, is that it has been found that CAFs and the immune cells in the TME can regulate each other’s functions via paracrine signaling pathways (41). Therefore, strategies targeting CAFs to enhance the infiltration and activation of T-cells could be effective to reverse the immunosuppressive TME and achieve good treatment outcomes for cancer patients. At present, the CAF-targeting therapeutic strategies in breast cancer could be achieved via multiple pathways, including blocking the activation of CAFs via TGF-β and Hedgehog signaling pathway inhibition, applying fibroblast-activation protein (FAP) vaccine, FAP-CART cells and inhibitors to target the activated CAFs, and targeting the different growth factors, chemokines, cytokines secreted by CAFs (83).

Overall, the remodeling of the immunosuppressive TME, by ablating the CAFs, reprogramming the activated CAFs, and targeting CAFs’ growth factor/cytokine production and secretion networks, hold great promise for effective breast cancer immunotherapy.

#### 3.2.2 Different biomarkers expressed by CAFs

The activated CAFs express some biomarkers at a significantly different level when compared to normal fibroblasts, such as α-SMA, vimentin, fibroblast-specific protein 1 (FSP1), FAP, caveolin-1, desmin, discoidin domain-containing receptor 2 (DDR2), and platelet-derived growth factor receptors alpha and beta (PDGFRα/β) (41, 84, 85). CAFs expressing different markers participate in different pathways (84, 85). For example, the FAP^+^/Vimentin^+^ CAFs could secret MMP1, collagen and TGF-β could modulate the ECM remodeling and tumor cell metastasis, while α-SMA^+^/FAP^+^/CD29^+^/PDPN^+^/PDGFRβ^+^ CAFs could secrete TGF-β and CXCL12 to affect tumor cell proliferation, migration and epithelial-mesenchymal transition (EMT) (83).

Some researchers have explored CAFs-targeted therapeutic strategies according to the specific biomarkers expressed on the CAFs (81, 86, 87). FAP, a membrane-bound serine protease, can act as a tumor targeting antigen, with relevant clinical drugs targeting FAP (81, 86, 87). However, since FAPs are extensively expressed in both tumor tissues and normal tissues [e.g., embryo (88), bone marrow (89) and placenta (90)], serious side effects or even death can be caused by systematic FAP-targeted therapy. As a result, biomaterial-based strategies targeting biomarkers expressed by CAFs have been explored for more effective cancer treatments, as compared with systematic biomarker-targeted therapies.

#### 3.2.3 Biomaterial-based strategies targeting CAFs for breast cancer treatments

Both biomaterial-based NPs and hydrogels have been explored to target CAFs for more effective breast cancer treatments. For instance, in order to selectively kill CAFs without leading to systemin toxicity, Zhen et al. developed a NP-based, FAP-targeted photo-immunotherapy that could selectively kill CAFs in breast tumors (81). Specifically, ZnF_16_Pc (a photosensitizer) was physically entrapped within ferritin (FRT, a compact nanoparticle protein cage), and a FAP-specific single chain variable fragment (scFv) was conjugated on the surface of FRT (81). This nanoparticle-based photoimmunotherapy (nano-PIT) approach could efficiently target and eliminate CAFs in breast tumors but exhibited little damage to healthy breast tissues. Further, CXCL12 secretion and ECM deposition was suppressed (81), and the tumor suppressive environment was reversed with enhanced cytotoxic T cell infiltration (81). Therefore, this photo-immunotherapy supplied a new approach for selectively targeting CAFs toward enhancing immunity and modulating the breast cancer TME to better treat breast cancer patients. Additionally, Sitia et al. engineered H-ferritin nanocages, loaded with navitoclax (Nav, a Bcl-2 inhibitor pro-apoptotic drug), could specifically bind to FAP^+^ CAFs in a mouse model of TNBC by conjugating the FAP antibody fragments on H-ferritin nanocages (91). Nav-loaded nanocages achieved efficient targeted delivery and yielded controlled drug release profile in FAP^+^ CAFs toward improving the breast cancer TME (91).

Moreover, CAF-targeted drug delivery nano-systems were also investigated in a xenograft mouse model of MCF-7 breast cancer (87). A cleavable amphiphilic peptide (CAP) containing a TGPA sequence that can be digested by FAP-α was designed to specifically respond to FAP-α expressed on the CAFs’ surfaces in tumor (87). CAP could self-assemble into fiber-like nanostructures in solution, while the self-assemblies can be transformed into stable drug-loaded spherical NPs (CAP-NPs), in the presence of hydrophobic chemotherapeutic drugs such as DOX, irinotecan (Iri) and paclitaxel (Tax) (87). It was found that cleavage enabled by FAP-α being expressed on the surface of CAFs, can rapidly disassemble the NPs to yield the efficient release of the encapsulated drugs specifically targeting at breast cancer sites. Such a transformer-based system can significantly increase local drug accumulation by disrupting the stromal barrier (87). Since FAP-α is commonly expressed and activated in most human tumors (92, 93), this FAP-α-targeted drug delivery nano-system has great application potential.

**Figure 3:**
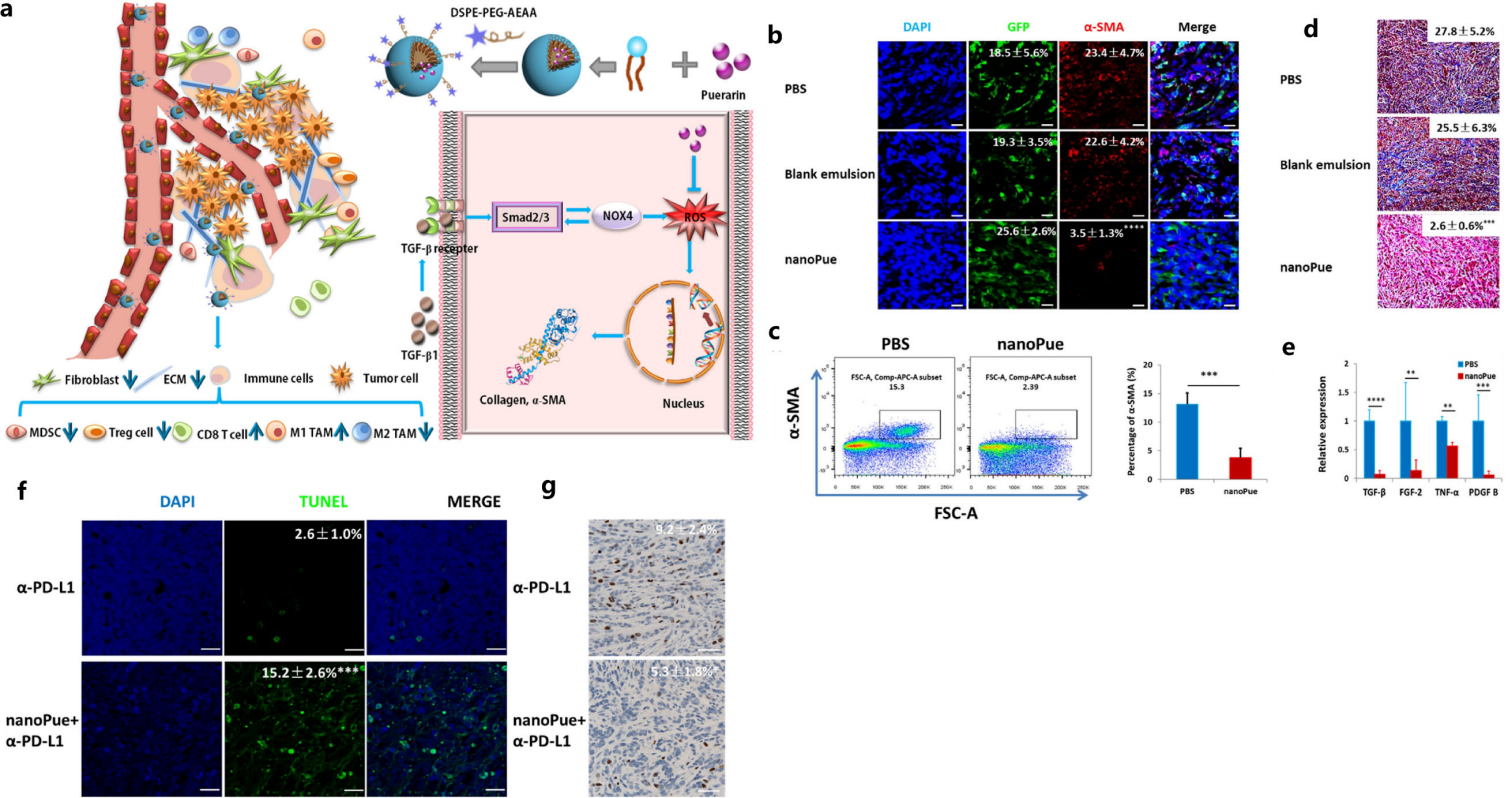
Illustration of TME remodulation by targeted puerarin delivery **(A)**, reduction of α-SMA positive TAFs in tumors by immunofluorescence staining **(B)** and flow cytometry analysis **(C)**, reduction of collagen by Masson’s trichrome staining **(D)** and TGF-β by RT-PCR **(E)**, higher apoptosis **(F)** and down-regulated Ki67 expression **(G)** by the nanoPue in combination with α-PD-L1. Reprinted with permission from Xu H, Hu M, Liu M, et al. (95). Copyright 2020, Elsevier. **p<0.01, ***p<0.001, ****p <0.0001.

Aside from NPs, hydrogel was also explored for regulating CAFs in 4T1 breast tumors. The peptide C16-GNNQQNYKD-OH (C16-N), which can self-assemble into supramolecular long filaments with hydrophobic cores, was co-assembled with losartan to form a hydrogel (94). This injectable peptide hydrogel inhibited the activity of CAFs, reduced the growth factor secretion of CAFs, and perturbed collagen synthesis. Further, it improved the efficacy of PEGylated DOX-loaded liposomes (Dox-L) toward inhibiting breast tumor growth, demonstrating the potential of an injectable hydrogel in regulating CAFs toward enhancing the chemotherapy effectiveness for breast cancer (94). In addition, puerarin nano-emulsion (nanoPue), with the ability of CAFs targeting by modifying with aminoethyl anis amide (AEAA) was developed. The AEAA-modified NPs were able to accumulate in CAFs and down-regulate reactive oxygen species (ROS) production to deactivate CAFs and improve the stromal microenvironment in murine TNBC model (with approximately a 6-fold reduction of CAFs in the nanoPue treatment group vs. control group), which also significantly enhanced the chemotherapy efficacy of paclitaxel (95). It was demonstrated that nanoPue effectively removed the intra-tumoral physical barrier formed by the dense ECM components produced by CAFs. As well, it increased the intra-tumoral infiltration of cytotoxic T cells and the activated immune activities in TME that further enabled nanoPue to synergize PD-L1 blockade therapy (**Figure 3**) (95).

Further, site-specific release of anti-breast cancer drugs, triggered by special proteins expressed in TME, has been investigated to remodel the immunosuppressive TME and improve the breast cancer immunotherapy efficiency. For instance, considering the high MMP2 expression level in breast cancer TME, Zhang et al. developed MMP2-sensitive lipid layer constructs, and demonstrated controlled release of LY3200882 (a TGFβ inhibitor) in MMP2-abundant TME (96). In addition, the combined release of LY3200882 and PD-L1 siRNA was achieved by the site-specific liposome-based delivery system to further enhance the anti-TNBC immunotherapy efficacy (96). The PD-L1 expression on CAFs decreased with the PD-L1 siRNA treatment, and LY3200882 caused reduced production of breast cancer ECM which promoted the nanomedicine and immune effector cells’ penetration into the tumor tissues (96). The dual inhibition of TGF-β and PD-L1 for both breast cancer cells and CAFs can effectively reverse the immunosuppressive TME for TNBC, suppress breast cancer cell proliferation and metastasis with minimal side effects, paving a new therapeutic strategy targeting breast cancer in the future (97).

Although some biomaterial-based strategies targeting CAFs for breast cancer treatments have shown some promise, there are still limitations. One of the significant challenges for targeting CAFs is their heterogeneity in the breast cancer TME and the lack of in-depth understanding of the specific biomarkers expressed, as well as signaling pathways triggered by the different CAF subtypes (41). As a result, clinical therapies targeting CAFs or the related signaling pathways sometimes do not show desirable treatment efficacy due to the presence of complicated tumor-restraining CAF subtypes that exhibit a suppression role in tumor progression (98–100). Thus, distinct therapeutic regimens could be considered for targeting the different subtypes of CAFs in the future breast cancer drug designs (80). Several biomarkers that have been regarded as being “specific” to CAFs, and utilized in previous anti-cancer drug designs, have recently been shown to be expressed on other cell types (36, 80). For example, FAPα is not only expressed on CAFs, but is also highly expressed in mesodermal cells (80). Similarly, α-SMA is not specific to CAFs either, but is abundantly present on normal fibroblasts, pericytes and smooth muscle cells (80). Those findings implied that anti-breast cancer therapies targeting those unspecific biomarkers can result in limited drug efficacy and future studies need to spend more efforts to identify the unique proteins or signaling molecules associated with the different CAF subtypes (36). Representative studies which have explored the use of biomaterials targeting CAFs for breast cancer treatments are summarized in **Table 2**.

**Table 2:** The use of biomaterials targeting cancer-associated fibroblasts in breast cancer.

Type of biomaterial	Effective components of the biomaterial	Biomaterial target	Effects	References
Ferritin nanoparticles	ZnF16Pc, anti-FAP scFv	CAFs	• Eliminate CAFs in tumors • Enhance T cell infiltration • Promote cancer cell death	(81)
H-ferritin (HFn) nanocages	Navitoclax (an experimental Bcl-2 inhibitor pro-apoptotic drug), anti-FAP fragments	CAFs	• Show specific targeting ability to induce CAFs’ apoptosis	(91)
Self-assembly nanoparticle	Doxorubicin (DOX), irinotecan (Iri) and paclitaxel (Tax), CAP (containing TGPA sequence which can be digested by FAP-α)	Cancer cells	• Achieve enhanced drug delivery and promising antitumor effects by responding to FAP-α expressed on the CAFs’ surfaces in tumor	(87)
Losartan-loaded C16-N hydrogel	Losartan	CAFs	• Improve the intratumoral accumulation and penetration of nanomedicine • Inhibit the CAFs and collagen I synthesis in orthotopic 4T1 tumors	(94)
Puerarin nano-emulsion (nanoPue)	Puerarin, DSPE-PEG-AEAA	CAFs	• Decrease ROS production to deactivate CAFs • Improve the stromal microenvironment in murine TNBC model • Enhance the chemotherapy efficacy of paclitaxel	(95)
Nanoparticle	PD-L1 siRNA, MMP2, cholesterol, LY3200882	CAFs and cancer cells	• Reverse the immunosuppressive TME for TNBC • Suppress breast cancer cell proliferation and metastasis	(96)

CAF, cancer-associated fibroblasts; FAP, fibroblast-activation protein; scFv, FAP-specific single chain variable fragment; CAP, cleavable amphiphilic peptide; C16-N, peptide C16-GNNQQNYKD-OH; DSPE–PEG–AEAA, DSPE–PEG–aminoethyl anis amide; ROS, reactive oxygen species; PD-L1, programmed cell death protein 1; MMP2, matrix metalloproteinase 2.

### 3.3 Myeloid-derived suppressor cells (MDSCs)

MDSCs are a class of bone marrow-derived inflammatory progenitor cells that play immunosuppressive roles in tumors. They are incapable of differentiating into dendritic cells, granulocytes, or macrophages (101). MDSCs could impair the normal function of effector immune cells to influence the TME (102).

#### 3.3.1 Interactions between MDSCs and breast tumor cells within TME

Release of breast tumor-derived inflammatory factors and chemokines within the TME can significantly stimulate MDSCs’ proliferation and infiltration in cancer tissues (103, 104). A series of proinflammatory cytokines such as interferon-γ (IFN-γ), IL-1β, IL-4, IL-13, PGE2, as well as STAT1, STAT6, nuclear factor-κB (NF-κB) signaling pathways all participate in MDSC activation (105). Once the MDSCs are activated, they could accelerate breast tumor growth and metastasis by generating more ROS, nitric oxide (NO) and immunosuppressive factors such as IL-10, impairing the anti-tumor immunity via deleting arginine and inhibiting CD8^+^ T cells (96, 106–108). Studies have shown the correlation between the MDSCs levels in peripheral blood/primary tumor tissues and the progression/metastasis status of breast cancer patients (109, 110). As a result, considering the MDSCs’ immunosuppressive characteristics, therapies targeting MDSCs’ recruitment, proliferation, and differentiation could be effective in terms of treating breast cancer patients.

#### 3.3.2 Biomaterial-based strategies targeting MDSCs for breast cancer treatments

Clinically, MDSCs-targeted strategies involved depletion, differentiation, modulation, and immunosuppressive activity inhibition of the MDSCs (105). In order to increase breast cancer immunotherapy efficacy, some researchers have developed biomaterials to improve the immunosuppressive TME via targeting MDSCs. Considering the fact that inflammatory reactions are often inevitable after surgical resection, Lu et al. developed hyaluronic acid (HA)-coated chitosan oligosaccharide-all-*trans*-retinoic-acid (COS-ATRA) micellar NPs, loaded with DOX (HA@CA/DOX) to modulate TME and alleviate the inflammatory responses after resection in 4T1 breast cancer model (104). In the HA@CA/DOX NP system, it was demonstrated that the hydrophilic COS and hydrophobic ATRA blocked the NF-κB pathway in breast cancer cells/MDSCs to reduce inflammation after resection, while HA shielded the excessive positive charge and enhanced breast cancer cell targeting via CD44 expressed on the cell surfaces (104). Additionally, ATRA eliminated the MDSCs not only in the breast tumor tissues, but also in the lung tissues after metastasis, thus inhibiting the formation of a pre-metastatic niche (PMN) (104). Moreover, to improve the MDSC deletion efficiency, they also developed NPs using low molecular-weight-heparin-all-*trans*-retinoic-acid (LMWH-ATRA) modified by a tumor targeting c(RGDfk) peptide and loaded with DOX, and immune adjuvant (α-galactosylceramide, αGC) (103). Such RLA/DOX/αGC NPs improved the immunosuppressive breast cancer TME, with αGC significantly enhancing the anti-tumor immunity in the breast and lung tissues (103). Specifically, it was found that the hydrophilic segment LMWH could tightly bind with P-selectin expressed on vascular endothelial cells (VECs), which inhibited MDSC recruitment, while the hydrophobic ATRA induced MDSC differentiation and depletion (103).

Indoleamine 2,3-dioxygenase (IDO), which is a rate-limiting enzyme of tryptophan catabolism, was revealed to be unregulated in tumor-infiltrating MDSCs and could hinder the anti-tumor immunity (109). Qiao et al. (111) developed a folated pH-degradable poly (vinyl alcohol) (PVA)-based nanogel (FA-NG) to simultaneously deliver docetaxel (DTX) and IDO1 inhibitor NLG919 (N9) to modulate the MDSCs’ infiltration in breast cancer tissues (111). For the FA-NG, PVA was modified with vinyl ether acrylate (VEA) groups for UV-crosslinking and degradation in response to low pH in TME (111). The study showed that FA-NG could be efficiently taken up by breast cancer cells followed by endo/lysosomal pH-triggered intracellular drug release, in which DTX induced cytotoxicity effects and immunogenic cell death (ICD), while N9 inhibited the IDO1-mediated immunosuppression and led to enhanced infiltration of CD8^+^ T cells/NK cells and reduced infiltration of MDSCs (111). This PVA-based degradable nanogel system provided a potential avenue for combining chemotherapy and immunotherapy to re-activate anti-tumor immunity in breast cancer treatment.

**Figure 4:**
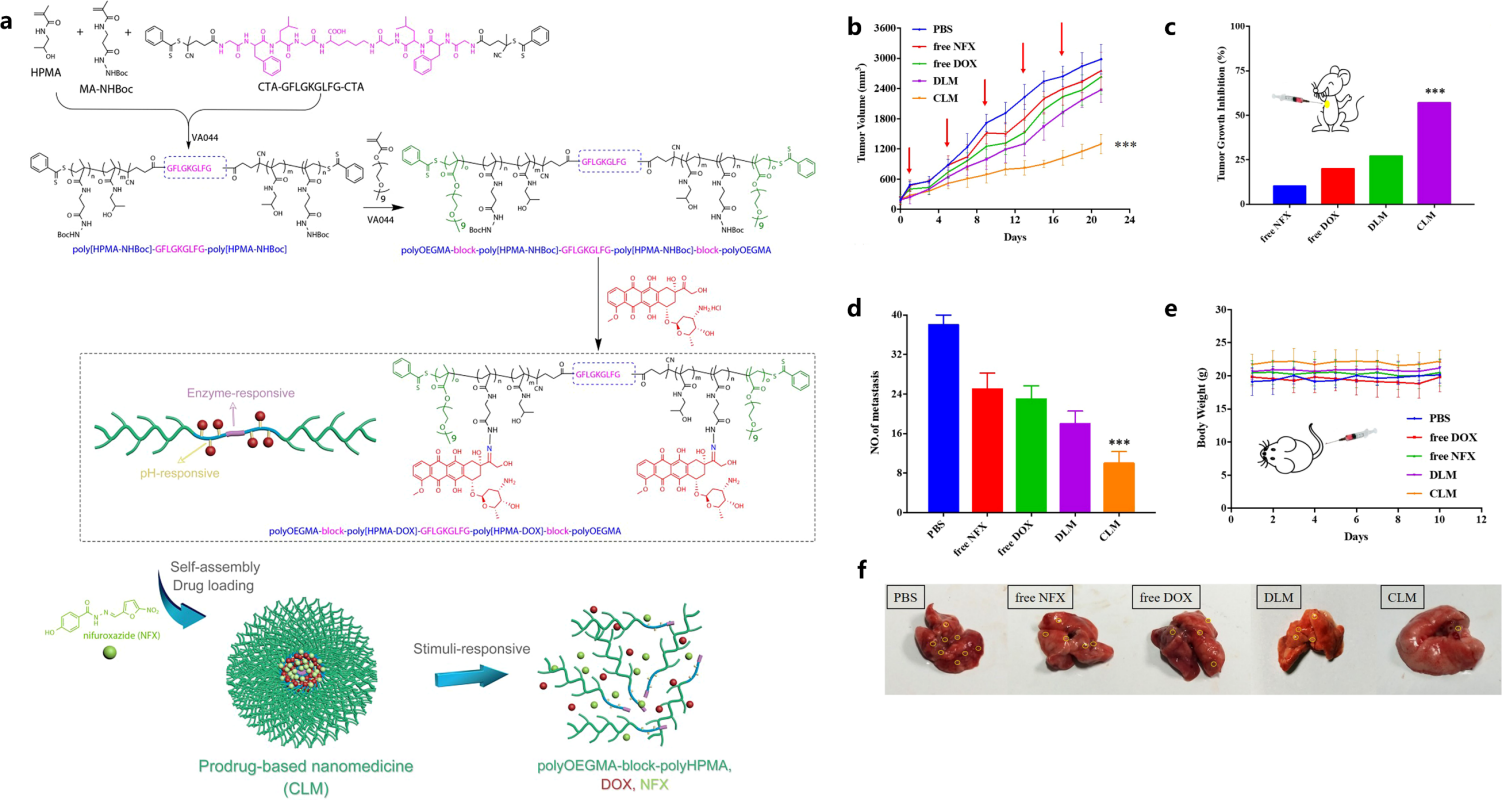
Illustration of CLM formation and stimuli-responsive drug release and degradation **(A)**, tumor growth curves **(B)**, tumor growth inhibition rate **(C)**, mean lung metastasis nodule numbers **(D)**, body weight changes **(E)**, visualized lung metastatic nodules **(F)**. Reprinted with permission from Luo L, Xu F, Peng H, et al. (112), Copyright 2019, Elsevier. ***p< 0.001.

Aiming at reducing MDSC infiltration in breast cancer and pulmonary metastasis, Luo et al. designed a cathepsin B/pH dual-sensitive block copolymer conjugated with DOX, which was then loaded with nifuroxazide (NFX) to self-assemble into co-prodrug-loaded micelles (CLM) (112). This micelle could be delivered to the tumor site, and DOX was released upon pH/enzyme stimuli, which caused tumor cell apoptosis, reduced expression of MMPs, and decreased MDSC infiltration (**Figure 4**) (112). DTX@VTX NPs (DTX: Docetaxel and VTX: VTX-2337 or Motolimod) with a core/shell structure were developed to address the immunosuppressive character of the breast cancer TME by depleting MDSCs and repolarizing the macrophages from M2-like phenotype to M1-like phenotype (113). Interestingly, it was found that the simultaneous application of DTX@VTX NPs with BMS-1 (a small-molecule PD-1/PD-L1 nano-inhibitor) NPs achieved synergistic chemo-immunotherapeutic anti-breast cancer effects when compared to the use of any single type of NPs (113).

Chemokine CCL2 is positively associated with increased numbers of M2-like macrophages and MDSCs (114). Liu et al. fabricated a targeted lipid-protamine-DNA (LPD) NP to deliver the plasmid DNA encoding CCL2 trap into TME, which can significantly reduce the numbers of M2-like macrophages and MDSCs in TNBC by blocking the CCL2/CCR2 signaling pathway (114). The LDP NPs was constructed by cationic protamine with plasmid DNA (pDNA) coated with DOTAP (1,2-dioleoyl-3- trimethylammonium propane chloride salt) liposome, and polyethylene glycol (PEG) was coated on the surface of LPD NPs, DSPE–PEG and the target ligand DSPE–PEG–AEAA were grafted onto the surface of liposomes. Specifically, the CCL2 trap could bind to CCL2 with a high affinity and specificity, which led to a significant reduction of CCL2 levels within breast cancer tissues, and increased infiltration of cytotoxic T cells (114).

Finally, it has been observed that complicated growth factor/cytokine/chemokine networks are responsible for regulating the proliferation, differentiation and activation of MDSCs, and that the latter cells are oftentimes heterogeneous populations that could modulate the TME via different mechanisms (115, 116). For example, in TNBC, monocytic-MDSCs (HLA^-^DR^−^CD33^+^CD14^+^) occupied predominant subsets when compared to granulocytic-MDSCs (HLA^-^DR^−^CD33^+^CD15^+^) (117). Therefore, different strategies based on MDSCs’ heterogeneity in distinct breast cancer subtypes could be considered in the future biomaterial-based immunotherapies targeting MDSCs. The representative studies that explored the use of biomaterials targeting MDSCs for breast cancer treatments are summarized in **Table 3**.

**Table 3:** The use of biomaterials targeting myeloid-derived suppressor cells in breast cancer.

Type of biomaterial	Effective components of the biomaterial	Biomaterial target	Effects	References
HA-coated COS-ATRA micellar nanoparticle	DOX, COS-ATRA, HA	Cancer cells, MDCSs	• Inhibit NF-κB activation • Promote MDSCs depletion to inhibit postoperative recurrence and lung metastasis of 4T1 breast cancer	(104)
RLA/DOX/αGC micellar nanoparticle	αGC, DOX, a tumor targeting c (RGDfk) peptide	VECs, MDSCs	• Induce MDSC differentiation and depletion • Improve the inflammatory and immunosuppressive microenvironment of the lung and tumor sites • Improve the anti-tumor immunity	(103)
Folated pH-degradable nanogels	Docetaxel, NLG919 (IDO1 inhibitor), Folic acid	Cancer cells	• Induce cytotoxicity effects and immunogenic cell death • Enhance CD8^+^ T cells/NK cells infiltration and reduce MDSCs infiltration • Reverse the IDO1-mediated immunosuppressive tumor microenvironment	(111)
Self-assembly micelles	Nifuroxazide, DOX, Cathepsin B/pH dual-sensitive block copolymer	Cancer cells, MDCSs	• Induce tumor cell apoptosis • Reduce MMPs expression • Decrease MDSC infiltration	(112)
DTX@VTX nanoparticle with a core/shell structure	DTX, VTX-2337	MDCSs, M2 macrophages	• Deplete MDSCs • Repolarize M2 macrophages to M1 phenotype • Increase number of cytotoxic CD8^+^ T cells	(113)
Liposome nanoparticle	CCL2 trap plasmid, protamine, DSPE-PEG, DSPE-PEG-AEAA	TAAs	• Suppress TAAs • Increase T cell infiltration • Reduce M2-like macrophages and MDSCs numbers in TNBC	(114)

MDSC, myeloid-derived suppressor cell; HA, hyaluronic acid; COS-ATRA, chitosan oligosaccharide-all-trans-retinoic-acid; DOX, doxorubicin; VECs, vascular endothelial cells; DTX, Docetaxel; VTX, VTX-2337 or Motolimod; CCL2, C-C Motif Chemokine Ligand 2; DSPE–PEG–AEAA, DSPE–PEG–aminoethyl anis-amide; TAAs, tumor-associated adipocytes; PLG, poly (DL-lactide-co-glycolide).

## 4 Biomaterials targeting other immune cells in TME to improve breast cancer immunotherapy

The number and composition of infiltrated innate and adaptive immune cells varies significantly in the TME of different breast cancer subtypes, and the infiltrated immune cell ratios could influence the anti-cancer immunotherapy outcomes (118, 119). Breast tumor infiltrating lymphocytes (TILs), such as helper T cells (CD3^+^/CD4^+^), cytotoxic T lymphocytes (CTL, CD3^+^/CD8^+^) and Tregs cells, were observed in breast tumors, and they were key indicators for breast tumor immunogenicity (107, 120). It was shown that high CD8^+^ T cells/Tregs ratios indicated favorable prognosis in primary breast tumors (121). Currently, facilitating the infiltration of beneficial immune cells to improve the immunosuppressive TME to achieve better breast cancer treatment efficiency remains a challenge. In the following, biomaterial strategies targeting other beneficial immune cells, including dendritic cells, natural killer (NK) cells, and T lymphocytic cells in the TME to improve breast cancer immunotherapies are discussed.

### 4.1 Dendritic cells (DCs)

Robust activation of tumor-specific antigen-presenting cells (APCs) warrants the efficiency of cancer immunotherapy (122, 123). DCs are representative APCs that are capable of capturing, processing, and presenting tumor antigens, and are crucial for inducing and regulating the innate and adaptive immune responses by activating T cells (124, 125). Thus, DCs typically play a positive role in inducing cancer immunity, and they could be classified into conventional DCs (cDCs), plasmacytoid DCs (pDCs) and monocyte-derived DCs (MoDCs), according to their ontogeny, phenotype and anatomical location (126). In TNBC, the ratio of cDCs:pDCs was higher when compared with other breast cancer subtypes (127) and it has been revealed that high levels of pDCs and cDCs are related with better survival outcomes for breast cancer patients (127, 128). It has also been reported that the abundancy of DCs is related to the sensitivity of chemotherapy drugs in breast cancer (129).

#### 4.1.1 DCs in breast cancer immunotherapy

It is known that DCs are vital targets in breast cancer immunotherapy and DCs-based vaccines have been developed as one of the main strategies to boost the anti-tumor immunity (130, 131). Currently, DC-based cancer vaccines mainly include MoDCs generated *in vitro*, DCs derived from CD34^+^ hematopoietic precursors, DCs isolated from the circulating blood, as well as allogeneic pDCs and pDC-derived exosomes (132). At present, DC-based immunotherapies used to potentiate host effector and memory CD8^+^ T cell responses have been widely studied and started to be applied clinically as DC vaccines (133). However, those different sources of DCs and DC-derived exosomes have shown limited anti-breast cancer efficacy in clinical applications due to DC population heterogeneity, high complexity of the breast cancer TME (133) and low DC targeting specificity for different subtypes of breast cancer tissues (132).

#### 4.1.2 Biomaterial-based DC strategies for breast cancer treatments

Recently, biomaterials have been explored to enhance the anti-breast-cancer effects of DCs. For example, Poly-lactide-co-glycolic acid (PLGA) is a biodegradable and relatively biocompatible material approved by FDA with great potential in vaccine delivery. PLGA NPs can enhance breast cancer antigen presentation (134). PLGA NPs that encapsulated tumor lysate antigens, derived from breast cancer tissues, can significantly enhance DC maturation and T cell immune response activation, with high targeting specificity, controlled release of antigen and improved antigen stability (135). At present, the main strategy for efficient and specific delivery of PLGA NPs to DCs within the TME include the coupling of NPs with monoclonal antibodies against DC specific receptors or natural ligands associated with DC endocytic receptors (e.g., mannan, a ligand for mannose receptor (MR) of DCs) (134). Mannan-incorporation of PLGA NPs resulted in their increased uptake by DCs vs. the non-modified control (134). Further, in breast tumor-bearing mice, Sanaz et al. investigated the effects of mannan-coupled PLGA NPs that contained both breast tumor cell lysate and poly-riboinosinic polyribocytidylic acid (poly I:C), which is a synthetic analog of dsRNA that can stimulate DC activation (124). It was found that simultaneous use of breast cancer cell lysate and DC-stimulating adjuvant in this PLGA NP system achieved efficient activation of DCs, with a large quantity of concomitant tumor associated antigens (TAAs) and C-type lectin receptor (CLR) ligands, causing elevated T cell responses and improved the breast cancer TME (124).

Antigen-based strategies targeting MRs of DCs showed some effectiveness in boosting the immunological effects of DCs in treating breast cancer. As well, other therapeutic avenues for DCs modulation were also exploited for breast cancer treatment. For instance, Wu et al. prepared the N-alkyl-PEI2k-LAC/SPIO nanocomposites that aided the transfection of siRNA targeting indoleamine 2, 3-dioxygenase (IDO) and enhanced MRI labeling efficiency in DCs (133). It was found that this N-alkyl-PEI2k-LAC/SPIO system minimized the degradability of exogenous siRNAs *in vivo* by loading and protecting those gene fragments, achieving the anti-tumor function by modulating DC vaccines (133). Additionally, chitosan-shelled nanobubbles (NBs) were functionalized with anti-CD1a antibodies to target DCs in breast cancer TME (136). The antiCD1a-functionalized NBs induced DC activation and enhanced their immune responses to inhibit tumor growth, showing great potential for breast cancer treatment (136). The representative studies that explored the use of biomaterials targeting DCs for breast cancer treatments are summarized in **Table 4**.

**Table 4:** The use of biomaterials targeting immune cells in breast cancer.

Type of biomaterial	Effective components of the biomaterial	Biomaterial target	Effects	References
Mannosylated PLGA nanoparticle	PLGA, TCL, poly I:C, mannan	DCs	• Activate DCs and induce tumor-specific T cell responses • Decrease tumor growth and metastasis	(124)
N-alkyl-PEI2k-LAC/SPIO nanocomposites	IDO siRNA	DCs	• Supply a highly efficient MR imaging platform for siRNA transfection into DCs	(133)
Chitosan-shelled nanobubble	DNA vaccine, anti-CD1a antibodies	DCs	• Transfect with high selectivity • Induce DC activation	(136)
PLGA-MnO_2_ nanoparticle	MnO_2,_ PLGA	tumor-produced hydrogen peroxide	• Enhance NK cells’ cytotoxicity	(137)
Acetylated dextran (Ace-DEX) microparticle	cyclic GMP-AMP	NK cells	• Increase natural killer cell numbers in TME • Result in NK and T cell-dependent anti-tumor immune response	(138)
DOPC-DPPC-mPEG2000-DSPE nanoparticle	cdGMP, MPLA	STING pathway and toll-like receptor 4	• Upregulate APCs and NK cells in the blood and tumor	(139)
Self-assembly nanoparticle	CD155 siRNA, anti-PD-L1 antibody	cancer cells	• Increase NK cell number • Reduce Tregs percentage • Inhibit cell growth and metastasis • Induce ICD	(140)
Liposome	CHI, BMS-202	cancer cells	• Improve immune cell infiltration • Boost T cell-mediated anti-tumor immunity	(141)
Micelle	PTX, anti-PD- L1 peptide	cancer cells	• Promote T cell infiltration • Increase tumor immuno-activating factors • Synergize PTX chemotherapy	(142)
ZrC nanoparticle	ZrC, PAH, BSA, FA	cancer cells	• Raise tumor-infiltrating cytotoxic T cells and helper T cells • Achieve lower dose PTT and RT to treatment	(143)
SPION nanoparticle	PD-1 siRNA, A2aR siRNA, cell-penetrating peptide HIV-1 TAT peptide	tumor- infiltrating T cells	• Suppress A2aR and PD-1 expression • Enhance T cells’ function	(144)

DC, dendritic cell; PLGA, polylactic-co-glycolic acid; TCL, tumor cell lysate; poly I:C, poly riboinosinic polycytidylic acid; NK, natural killer cells; MnO_2_, manganese dioxide; cdGMP, cyclic diguanylate monophosphate; MPLA, mono-phosphoryl lipid A; STING, stimulators of interferon genes; APC, antigen-presenting cell; DOPC, 1,2-dioleoyl-sn-glycero-3-phosphocholine; DPPC, 1,2-dipalmitoyl-sn-glycero-3-phosphocholine; mPEG2000-DSPE, methoxy-poly(ethylene glycol)-2000 1,2-distearoyl-sn-glycero-3-phosphoethanolamine-N; cdGMP, cyclic diguanylate monophosphate; MPLA, mono-phosphoryl lipid A; ICD, immunogenic cell death; CHI, chidamide; PTX, paclitaxel; PAH, polyallylamine hydrochloride; BSA, bovine serum albumin; FA, folic acid; RT, radiation therapy; PTT, photothermal therapy; SPION, superparamagnetic iron oxide.

### 4.2 Natural killer (NK) cells

NK cells, which belong to the innate lymphoid cell family (145), are derived from the multipotent CD34^+^ hematopoietic progenitors in bone marrow and they mature in the bone marrow and lymphoid organs (146). As a type of natural cytotoxic lymphocyte, they can recognize and kill MHC I-low or MHC I-negative breast cancer cells that have escaped from CD8^+^ cells with minimal damage to healthy tissues due to their expression of specific stimulatory and inhibitory receptors (145, 147). Therefore, NK cells are important anti-tumor agents to be considered in cancer immunotherapy (145, 148).

Human tissues mainly consist of two NK subpopulations (CD56^bright^ and CD56^dim^) with different functional and metabolic characteristics (149). 80%-90% of CD56^dim^ NK cells are present in human blood, bone marrow, lung and spleen, and only less than 20% of them exist in the lymph nodes (149). Allogeneic NK cells have great potential for cancer immunotherapy because they can be easily accessed from peripheral blood, umbilical cord blood or post-partum placenta (150). In addition, it was found that gap junctions, membrane nanotubes and exosomes are key mediators enabling the effective intercellular communications between NK cells and cancer cells, which are critical for the accumulation of ions, proteins and cytokines in cancer cells, enabling the induction of their apoptosis or necrosis (151).

#### 4.2.1 Interactions between NK cells and other immune cells within the TME

Moreover, cross-talk between NK cells and other immune cells enables the cooperation between them in order to regulate their anti-tumor immunity (152–154). Some factors produced by NK cells, such as IFN-γ, CCL5, XC-chemokine ligand 1 (XCL1) and FMS-related tyrosine kinase 3 ligand (FLT3L) could interact with T cells and DCs in the TME to enhance the T cell responses, and NK cells could also directly kill the inhibitory MDSCs (152–154). Immunosuppressive TME played a negative role for NK cell activation and limited their anti-tumor effects by disturbing their energy consumption and metabolism patterns (148). Some soluble factors in the TME, such as TGF-β, prostaglandin E_2_, _L_-kynurenine and picolinic acid, could reduce NK cells’ proliferation, activation and cytotoxicity effects (150). What’s more, Tregs can also inhibit NK cell function via TGF-β-related signaling pathways (155, 156). Therefore, modulating the abundance and activation of NK cells at tumor sites is critical for effective anti-tumor treatments. It has been well-acknowledged that the NKs’ anti-tumor activity can be modulated via altering the balance of stimulatory and inhibitory signals in the TME, including cytokines/receptors’ expression patterns (157).

#### 4.2.2 Biomaterial-based NK strategies for breast cancer treatments

Since the hypoxic and nutrient-lacking TME could suppress NK cell metabolism and their cytotoxic function (158), improving TME hypoxia with the help of biomaterials has been investigated to enhance the potency of NK cells. For instance, PLGA-encapsulated-MnO_2_ NPs were designed to sustain high oxygen level in the core of cancer spheroids, in order to boost NK cell cytotoxicity (137). Specifically, the MnO_2_ NPs were used to catalyze the degradation of tumor-produced H_2_O_2_, producing oxygen in the TME. It was found that PLGA encapsulation endowed MnO_2_ NPs with improved biocompatibility, enabled a first-order oxygen production profile and sustained high oxygen tension when compared to bare MnO_2_ NPs, in the presence of H_2_O_2_ (137).

To enhance the expansion and activation of NK cells in tumor sites, degradable biomaterials were explored to deliver immune stimulating molecules and improve the anti-breast tumor immune responses. For example, acetylated dextran (Ace-DEX) microparticles (MPs) were designed to deliver pathogen associated molecular patterns (PAMPs) to induce immune response (138). Cyclic GMP-AMP (cGAMP) MPs exhibited the most effective anti-tumor efficacy, causing an increase in NK cell number in the TME. These MPs activated NKs and T cells-dependent anti-tumor immune responses and exhibited significant tumor inhibition effects in both melanoma and TNBC models (138). In other work, an immuno-NP made of 1,2-dioleoyl-sn-glycero-3-phosphocholine (DOPC), 1,2-dipalmitoyl-sn-glycero-3-phosphocholine (DPPC) and methoxy-poly (ethylene glycol)-1,2-distearoyl-sn-glycero-3-phosphoethanolamine-N (mPEG2000-DSPE) was investigated for treating breast cancer (139). Cyclic diguanylate monophosphate (cdGMP, an agonist of the STING pathway) and mono-phosphoryl lipid A (MPLA, a toll-like receptor 4 agonist) were both encapsulated into the DOPC-DPPC-mPEG2000-DSPE NPs, which significantly upregulated the expression of IFN-β and boosted the activity of APCs and NK cells in blood, and tumor tissues in the TNBC model (139). When considering the high expression pattern of PD-L1 and CD155 in TNBC cells, Chen et al. developed the PD-L1 and CD155 asynchronous blockade NP system (140). Specifically, CD155 siRNA (siCD155) was loaded in mPEG-PLGA-PLL (PEAL) NPs coated with PD-L1 blocking antibodies (P/PEAL_siCD155_), which modulated CD155-mediated immune surveillance in the immune escape TNBC model, causing increased NK cell number, reduced percentage of Tregs, significant inhibition of breast cancer cell growth and metastasis and induction of ICD (140). The representative studies that explored the use of biomaterials targeting NKs for breast cancer treatments are summarized in **Table 4**.

### 4.3 T lymphocyte cells

T lymphocyte cells refer to the immune cells that are derived from bone marrow and mature in thymus. These cells could specifically recognize antigens via the TCR on their cell surface in order to exhibit robust lethality for infected cells (159). The infiltration of CD8^+^ T cells is associated with breast cancer patient survival (160), while CD4^+^ T helper cells also play a critical role in anti-tumor immune response (161). Immune checkpoints and receptors, which exhibit their suppressive role to inhibit or exhaust T lymphocyte cells and prevent the potential damage from excessive immune responses, are promising immunotherapeutic targets in cancer treatment (101, 162). These include PD-1 and its ligand PD-L1, cytotoxic T lymphocyte antigen 4 (CTLA-4), T cell immunoglobulin and mucin domain-containing 3 (TIM3), IDO, lymphocyte activation gene 3 (LAG3) and V domain Ig suppressor of T cell activation (VISTA) (101, 162). PD-1, which acted as a suppressive factor in TME, is a potent therapeutic target in breast tumor immune therapy (163). The interactions between PD-1 (expressed on tumor-infiltrating T cells surface) and PD-L1 (expressed on the cancer cells or antigen-presenting cells), negatively regulate the activation of cytotoxic T cells and can lead to T cell exhaustion and suppress the immune responses against tumor cells (164). Therefore, targeting PD-1 or PD-L1 could effectively improve the immunosuppressive TME. The antibodies that bind to the above inhibitory receptors or ligands to enable T cell activation could enhance the anti-tumor activity.

Currently, several immune checkpoint-targeting antibodies have been approved by the FDA, such as monoclonal antibodies pembrolizumab (KEYTRUDA) and nivolumab (OPDIVO, anti-PD-1), atezolizumab, avelumab, durvalumab (anti-PD-L1) and ipilimumab (anti-CTLA-4), which could increase overall survival of patients for different cancers (162, 165). However, the different immune status of patients and their specific immune checkpoint blocker expression levels can affect the treatment efficacy for immune checkpoint targeting antibodies (166).

In addition to the traditional approaches that indirectly promote the activation of T cells, biomaterial-based breast cancer immunotherapies which stimulate proliferation, differentiation, activation and infiltration of T cells and induce the tumor-specific T cell responses have been explored.

#### 4.3.1 Biomaterial-based strategies targeting T cells for breast cancer treatments

At present, biomaterials have been used to design some antibody-free molecules targeting for PD-1/PD-L1 blockade and T cell-mediated anti-tumor immunity, for enhancing the effectiveness of breast cancer treatments. For example, a liposome system was designed to co-deliver chidamide (CHI, a novel subtype-selective histone deacetylase inhibitor) and BMS-202 (a small-molecule PD-L1 inhibitor) in order to enhance T-cell recognition for treating TNBC (141). CHI, the epigenetic modulator, increased the expression level of PD-L1, MHC I and MHC II on cancer cells and improved the immune cell infiltration by inducing tumor cell apoptosis and ICD (141). The steady release of BMS-202 in tumors induced the dimerization of PD-L1 to inhibit the interaction of PD-1/PD-L1 and boosted the T cell-mediated anti-tumor immunity. This combined treatment of epigenetic regulation and immune checkpoint blockade (ICB) for TNBC exhibited effective inhibition of breast tumor growth and metastasis (141). Although ICB is an efficient treatment strategy for cancer, inflammatory side effects, which are termed immune-related adverse events including vitiligo, encephalitis, pneumonitis and colitis, can often occur clinically (165). Additionally, some chemotherapeutic agents have been reported to cause the overexpression of PD-L1 on cancer cells and inhibit the activation of CD8^+^ T cells at tumor sites, which can assist the cancer cells to evade the T cell immune surveillance, since the deletion of PD-L1 can activate the function of CD8^+^ T cells, indicating the combination of chemotherapy and ICB could exhibit promising treating outcome for cancer patients (167).

**Figure 5:**
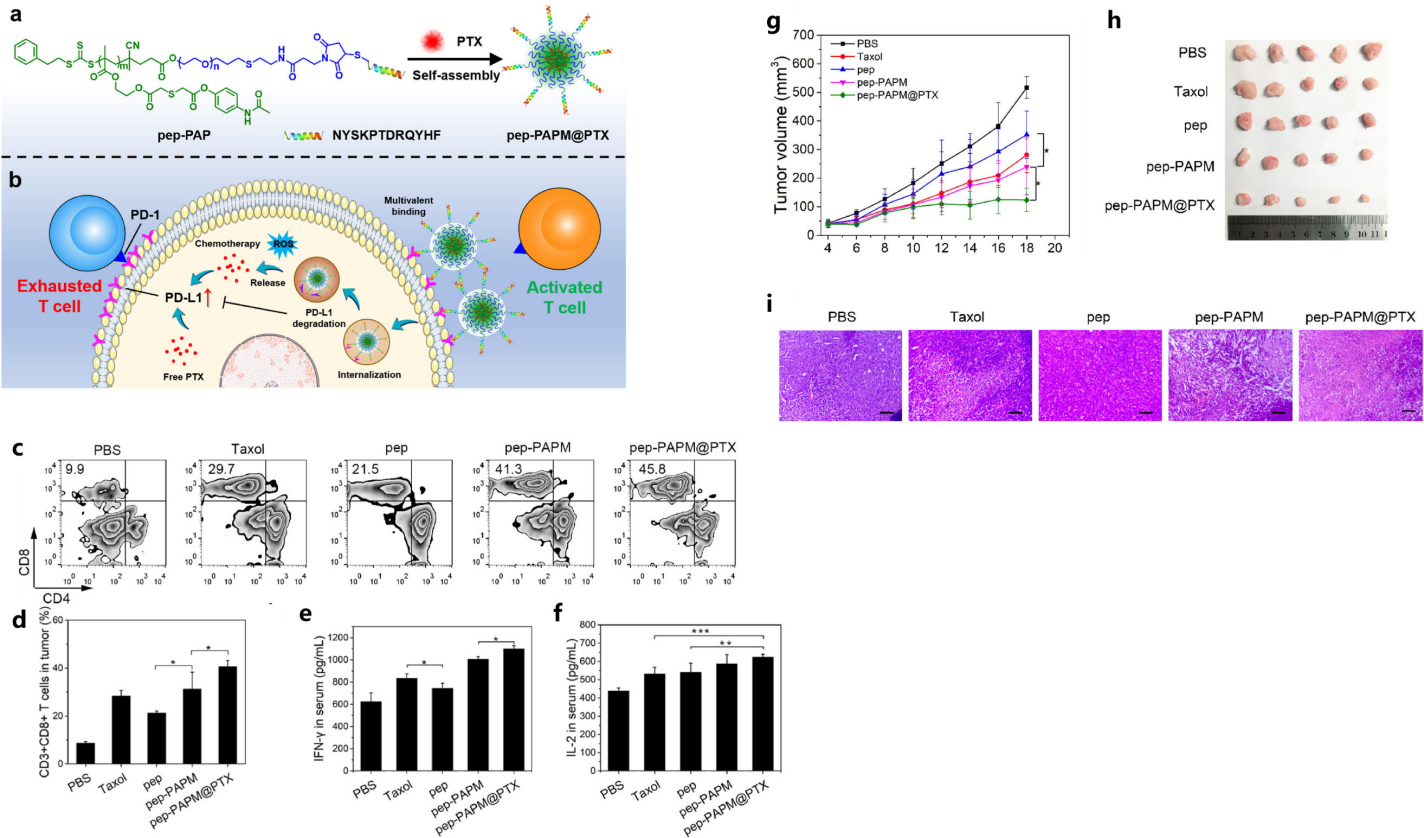
The illustration of pep-PAPM@PTX micelle **(A)**, schematic illustration of the ROS-responsive synergistic delivery system **(B)**, pep-PAPM@PTX micelle increased CTLs’ infiltration and contents of inflammatory cytokines **(C-F)**, tumor volume changes **(G)** and pictures of resected tumors **(H)**, H&E staining of tumors **(I)** in different groups. Reprinted with permission from Hu D, Zhang W, Xiang J, et al. (142). Copyright 2022, Elsevier. *p<0.05, **p<0.01, ***p<0.001.

Hu et al. developed a ROS-responsive synergistic delivery system (pep-PAPM@PTX) that combined immunotherapy (ICB therapy) and chemotherapy in a TNBC model (142). This micelle integrated physically-encapsulated paclitaxel and surface-modified anti-PD-L1 peptide, in which the PD-L1-targeting D-peptide on the micelle surface could multivalently bind the PD-L1 on breast cancer cell surface and downregulate PD-L1 by driving it into lysosomal degradation, and then alleviate its immunosuppression to cytotoxic T cells (142). What’s more, the micelle could respond to elevated ROS levels by releasing PTX, and this micelle-mediated combined therapy exhibited significant efficacy in TNBC model, by improving the immune-microenvironment with the enhanced T cell infiltration and increased release of tumor immune activation factors (**Figure 5**) (142).

In addition, conventional biomaterial-based NPs such as ZrC NPs were used in photothermal and radiotherapies for breast cancer treatment (143). Firstly, ZrC NPs were modified by bovine serum albumin (BSA) and folic acid (FA) to improve the overall biocompatibility of the particles, as well as their tumor-targeting capability. The engineered ZrC NPs possessed radiosensitizer and photosensitizer potential without obvious systemic toxicity, and endowed more curative efficiency for radiation therapy (RT) and photothermal therapy (PTT) in breast cancer treatment (143). This strategy resulted in improved immune responses by raising the tumor-infiltrating cytotoxic T cells and helper T cells, and also achieved lower dose PTT and RT for TNBC treatment and minimized the associated side effects (143).

Furthermore, strategies for increasing the proliferation and anti-tumor activity of T cells were also investigated. A2aR is one of the adenosine receptors and its inhibition could improve the T cell anti-tumor activity (168, 169). SPION (superparamagnetic iron oxide)-chitosan lactate (CL)-TAT NPs loaded with PD-1/adenosine-A2A receptor (A2aR) specific siRNA combined with DC vaccines were used for breast cancer treatment (144). This combined therapy efficiently delivered the siRNA to tumor-derived T cells at the breast tumor site and then suppressed PD-1 and A2aR expression. On the other hand, the DC vaccination significantly increased the anti-tumor responses in A2aR^−/−^ and PD-1^−/−^ T cells (144). It was also noted that IL-17 and IFN-γ secretion, T cell proliferation, and anti-tumor activities were enhanced, while the breast tumor growth, metastasis and angiogenesis were inhibited (144). The representative studies that explored the use of biomaterials targeting T cells for breast cancer treatments are summarized in **Table 4**.

## 5 Discussion and future perspectives

Breast cancer is one of the most prevalent malignancy worldwide and its distal metastasis (such as lymph nodes, lung, bone, liver) can seriously threaten the health and life quality of patients and significantly lower their survival rate (170). Although there are various subtypes of breast cancer, TNBC has been identified to be the most aggressive form with limited treatment options due to its unique molecular characteristics (171). The main purpose of breast cancer therapy is to eradicate the primary tumor tissue, inhibit the growth and metastasis of cancer cells, and avoid the risks of breast cancer recurrence (172). Traditional therapies for breast cancers, such as chemotherapy, radiotherapy and photodynamic therapy could directly kill cancer cells accompanied with the cancer-associated ICD and lead to the release of antigens to initiate immune responses (57, 141, 173). However, these traditional strategies also have some unavoidable drawbacks such as the non-specific killing of normal proliferating cells within the breast tissue or in the neighboring tissues and patients who are diagnosed at an advanced stage appear to be resistant to the traditional strategies (173). As a result, a more immunogenic TME needs to be induced to precisely attack the tumor cells, appreciating that the immune-suppressive TME is a major barrier for effective breast cancer therapy (174, 175). Recently, it has been noted that delivering effective amounts of immunostimulatory agents/tumor antigens into immune cells is critical for inducing robust immune response against breast cancer. However, insufficient transfer of breast tumor antigens and adjuvants into desired regions (e.g., lymph nodes, APCs), as well as enzymatic degradation of those molecules are significant obstacles of typical breast cancer immunotherapies.

Over the most recent few years, biomaterials have been designed and applied for mainly improving breast cancer immunotherapy efficacy and minimizing the anti-cancer side effects (12). Biomaterial-based NPs have been designed as anti-breast cancer drug carriers due to their biocompatibility, lower toxicity and the relatively small sizes that allow them to be easily taken up by the breast cancer cells (124). In addition to being anti-cancer drug carriers, since NPs can be designed to be selectively accumulate in breast tumor tissues, they can also act as imaging agents as well (176). Targeting breast cancer TME has become a promising strategy for breast cancer treatments. The inhibitory factors and inflammatory cells in the TME can promote breast tumor growth by reducing anti-tumor responses (144).

Multiple studies have indicated that biomaterials could play important roles in breast cancer treatments, including modulating the M2 TAMs, CAFs, MDSCs and activating DCs, NKs, and multiple T cell types, as discussed in Sections 3&4. Although biomaterial-based strategies targeting those cell types have shown some promise, there are still limitations. For example, one of the significant challenges for targeting M2-TAMs and CAFs is their significant heterogeneity in the breast cancer TME (84, 177) and the lack of in-depth understanding of the specific biomarkers expressed, and signaling pathways triggered by the M2-TAMs and different CAF molecular subtypes (41). Specifically, the biomarkers for M2-TAMs and CAFs may lack specificity and sensitivity due to their population heterogeneity (84, 177). As a result, clinical therapies targeting M2-TAMs and CAFs, or their related signaling pathways sometimes do not show desirable treatment efficacy due to the presence of complicated tumor-restraining M2-TAMs and CAF subtypes (98–100). Future biomaterial-based therapeutic strategies need to be designed to target specific biomarkers expressed by the M2-TAMs and different CAF subpopulations in the breast cancer tissues (80, 178, 179) in order to advance more effective treating outcomes.

Additionally, it has been observed that complicated growth factor/cytokine/chemokine networks are responsible for regulating the proliferation, differentiation, and activation of MDSCs, and the latter are oftentimes heterogeneous populations in different cancer subtypes and could modulate the TME via different mechanisms (105, 116). For example, in TNBC, monocytic-MDSCs (HLA-DR^−^CD33^+^CD14^+^) occupied the predominant subsets compared to granulocytic-MDSCs (HLA-DR^−^CD33^+^CD15^+^) (117). Therefore, different strategies based on MDSCs’ heterogeneity in distinct breast cancer subtypes could be considered in future biomaterial-based immunotherapies targeting MDSCs.

Further, within the breast cancer TME, it was also found that the DCs play a vital role in initiating and regulating antigen-specific immune responses by acting as the sentinels of the immune system (129). Recently, Zhao et al. revealed new biomarkers of DCs for the breast cancer treatment and prognosis, including *BCL9* (*B-cell lymphoma 9)*, *TPR* (tetra-tripeptide repeat), and *RBBP5* (*Retinoblastoma-binding protein 5*) (129). For the first time, the group found that *HNRNPU* and *PEX19* were closely related with the prognosis of DCs (129). Future studies can consider those biomarkers as potential targets in designing biomaterial-based drug delivery systems for breast cancer immunotherapy. Additionally, biomaterial-based strategies targeting NKs/multiple T cell types showed some promises, however, there is still lack of deep understanding for the molecular signaling pathways underlying the interactions between NKs, or different types of T cells, with the breast tumor cells and other types of tissue cells within the breast tumor niche environment. Therefore, future studies need to spend more effort on revealing the detailed interaction networks involving NKs, various T cell types, breast tumor cells, cancer stem cells, epithelial cells and fibroblasts etc.

Recently, it was also found that tumor-associated adipocytes can play a vital role in the TME of TNBC tissues and promote breast tumor growth and metastasis (114). NPs have been designed to inhibit tumor-associated adipocytes to remodel the TME of TNBC (114). However, studies using NPs to target tumor-associated adipocytes for TNBC treatment are still very limited. Future studies are needed to more deeply investigate the mechanisms involved in the remodeling of the breast cancer TME by the tumor-associated adipocytes, and to enable more effective biomaterial-based strategies targeting tumor-associated adipocytes.

**Figure 6:**
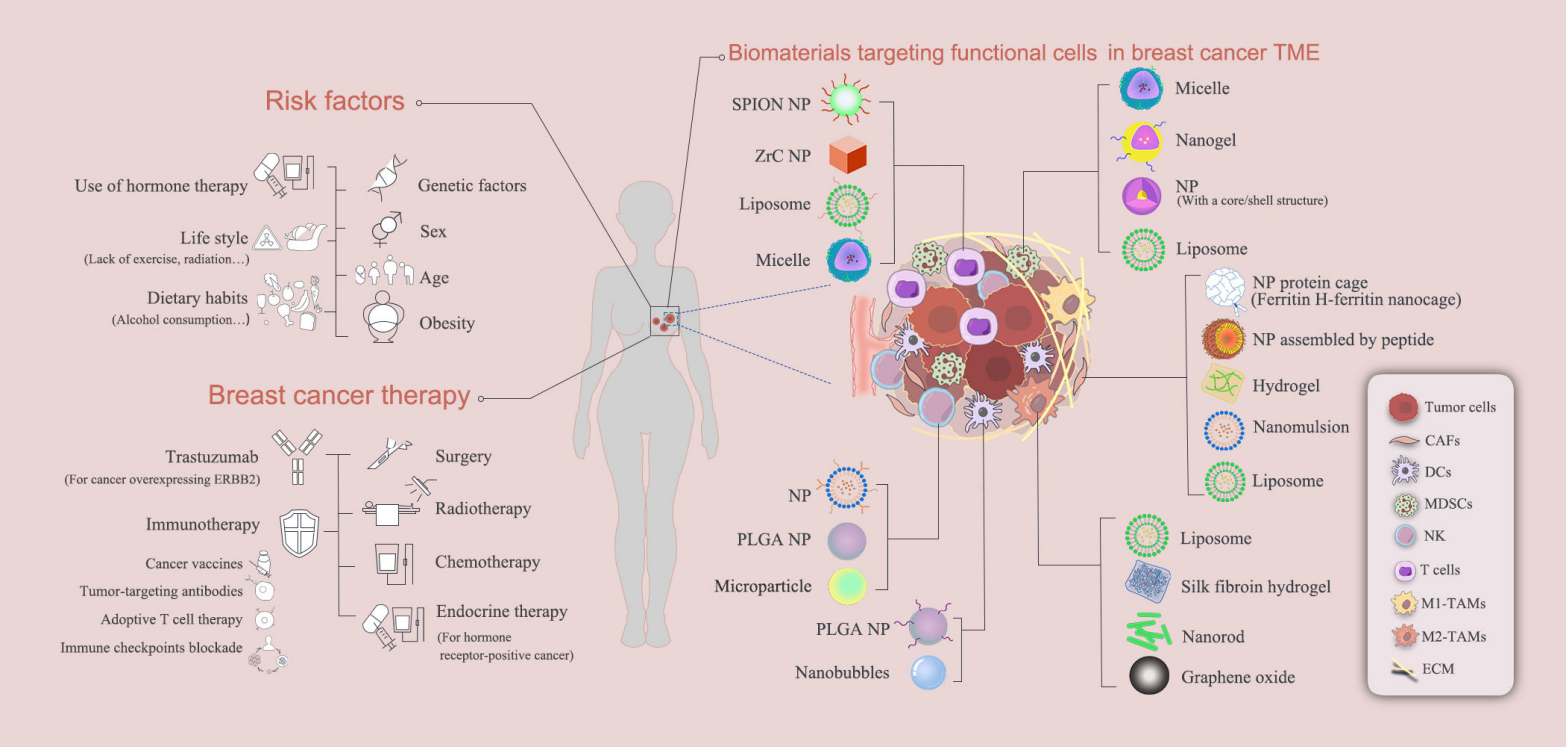
Biomaterial-assisted treatment therapies targeting breast cancer.

Biomaterial-based immunotherapies targeting breast cancer TME have shown some effectiveness in treating breast cancer patients. However, complete eradication of breast cancer, especially the TNBC, still remains a great challenge. As multiple cell types (e.g., breast cancer cells, epithelial cells, M2 TAMs, CAFs, MDSCs, DCs, NKs and multiple T cell types) are involved in the breast cancer TME and they share intertwining signaling mechanisms controlling the breast cancer development and progression, future biomaterial-based strategies need to consider those different cell types and their interaction mechanisms, to enable more effective treating outcomes. Several of the biomaterial-assisted treatment therapies discussed above are depicted in **Figure 6**.

Since suppressive TME hinders effectiveness of anti-breast cancer immunotherapy, nanomaterials have been designed to be used in combination with anti-breast cancer drugs (e.g., mRNA drugs) to activate immune cells within the breast cancer TME to achieve enhanced biocompatibility and targeting specificity. However, efficient permeation and accumulation of cargo-loaded-NPs in breast cancer TME are still challenging to accomplish. Both physical and chemical properties need to be well-considered for the nanomaterials designed for anti-breast cancer applications. Specifically, in terms of physical properties of the nano-biomaterials, the shape and structure of the NPs (Spiky, Spherical DNA or RNA, polyhedral, sphere-nanosheet shape transitions etc.), size of NPs (100-1000 nm), surface charge of NPs (cationic, anionic, zwitterionic), mechanical strength of NPs (such as elastic modulus) as well as the hydrophobicity/hydrophilicity of the NPs can all directly or indirectly affect the functions of immune cells and immune responses within the breast cancer TME (180). Additionally, the chemical properties of nano-biomaterials such as possession of specific functional groups or bioactive ingredients can influence the biomaterials’ biocompatibility, biodegradability, specific interactions with immune cell types and endocytosis efficiency (181). Therefore, future biomaterial designs for anti-breast cancer immunotherapies need to put great emphasis on developing biomaterials with both appropriate physical and chemical properties that can be biocompatible with the drugs (e.g., mRNA) and the breast cancer TME, and more importantly, can efficiently penetrate into the desired immune cells (e.g., T cells, dendritic cells, NK cells) to load the immunostimulatory agents/tumor antigens/anti-breast cancer drugs (e.g., mRNA), in order to activate the immune cells for enhanced anti-breast cancer activities.

In addition, novel receptors that are overexpressed in the different subtypes of breast cancer need to be identified in future research and new nano-biomaterials can be designed to have surface modifications according to the unique expression pattern of those novel receptors to achieve specific and efficient interactions between cargo-loaded nano-biomaterials and breast cancer cells to boost the effectiveness of the anti-breast cancer treatment. For instance, a recent study designed a self-assembled nanoplatform named GENP by using amphiphilic molecule of stearic acid-modified EGFR-targeting peptide GE11 with DOX and GENP exhibited high loading efficiency and sustainable DOX release (182). GENP itself suppressed TNBC cell proliferation via EGFR-downstream PI3K/AKT signaling pathways, contributing to the synergistic treatment with its drug release (182). In addition to design nano-biomaterials that can target specific receptors expressed within breast cancer tissues, biomaterials that are responsive to the specific physiological stimuli (pH, enzymes and redox potential) of the breast cancer TME and exogenous energetic stimuli (light, magnetics and ultrasound) could be designed (183) for breast cancer treatments.

Liposomes could exhibit adjuvant effects for breast cancer treatments by regulating the accumulation and release of cargos to elicit host immunity (184). Previous study has demonstrated that both cationic and anionic liposomes with good biocompatibility could exhibit highly-efficient adjuvant effect in colorectal cancer treatment (185). Those liposomes functioned by mobilizing DCs via the MyD88-TRAF6 pathways, which could further activate T helper cells and CD8^+^ T cells and enhance host immunity by regulating Th1, Th2 and Tregs (185). In addition, a library of antigens and adjuvant-free liposomes with variable surface charges (via changing the composition of cationic, anionic and zwitterionic lipids) has been developed (185). With those interesting findings, it can be seen that an alternative strategy that the field can pursue for future anti-breast cancer treatments is utilizing liposomes (e.g., anionic, anionic) to enhance DC maturation and mobilize the immune responses in the TME.

Further, with in-depth research of the literature, it was found that more anti-breast cancer drugs/biosimilars have been developed recently and they have been approved to work alone or in combination with others to target different breast cancer subtypes (**Table 5**). For example, ENHERTU (fam-trastuzumab deruxtecan-nxki) has been approved to be used in patients with unresectable or metastatic HER2^-^ breast cancer, while the combination of Abemaciclib with endocrine drugs can be used for adjuvant treatment of HR^+^HER2^-^ breast cancer. Further, it was found that the triple combination of Inavolisib, palbociclib and fulvestrant could treat HR^+^HER2^-^, locally advanced or metastatic breast cancer. With the success of using multiple anti-breast cancer drugs simultaneously, it can be expected that future studies can design nano-biomaterials that can efficiently incorporate multiple types of anti-breast cancer drugs to further enhance targeted delivery to the breast cancer TME and boost immunotherapeutic treatment outcomes.

**Table 5:** FDA-approved drugs/biosimilars used in the different types of breast cancer.

Date of approval	Drugs’/biosimilars’ name	Treatment strategy	Breast cancer subtypes	Trial information
Oct-2024	Inavolisib (Itovebi, Genentech, Inc)	Combined with palbociclib and fulvestrant	HR^+^, HER2^-^, locally advanced or metastatic breast cancer	INAVO120 (NCT04191499)
Sep-2024	Ribociclib (Kisqali, Novartis Pharmaceuticals Corporation)	Combined with an aromatase inhibitor for the adjuvant treatment	HR^+^, HER2^-^ stage II and III early breast cancer	NATALEE (NCT03701334)
Nov-2023	Capivasertib (Truqap, AstraZeneca Pharmaceuticals)	Combined with fulvestrant	HR^+^, HER2^-^ locally advanced or metastatic breast cancer	CAPItello-291 (NCT04305496)
Feb-2023	Sacituzumab govitecan-hziy (Trodelvy, Gilead Sciences, Inc)	——	HR^+^, HER2^-^ (IHC 0, IHC 1^+^ or IHC 2^+^/ISH^-^) breast cancer	TROPiCS-02 (NCT03901339)
Jan-2023	Elacestrant (Orserdu, Stemline Therapeutics, Inc)	——	ER^+^, HER2^-^, ESR1-mutated advanced or metastatic breast cancer	EMERALD (NCT03778931)
Aug-2022	Enhertu (fam-trastuzumab deruxtecan-nxki)	——	HER2-low breast cancer	DESTINY-Breast04
May-2022	Enhertu (fam-trastuzumab deruxtecan-nxki)	——	HER2^+^ breast cancer	DESTINY-Breast03
March-2022	Olaparib (Lynparza, AstraZeneca Pharmaceuticals, LP)	Adjuvant treatment	HER2^-^ high-risk early breast cancer	OlympiA (NCT02032823)
Oct-2021	Abemaciclib (Verzenio, Eli Lilly and Company)	Combined with tamoxifen or an aromatase inhibitor for adjuvant treatment	HR^+^, HER2^-^, node-positive, early breast cancer	MonarchE (NCT03155997)
July-2021	Pembrolizumab (brand name Keytruda)	Combined with chemotherapy as neoadjuvant treatment	High-risk, early-stage, TNBC	KEYNOTE-522
April-2021	Sacituzumab govitecan-hziy (Trodelvy, Immunomedics Inc)	——	Advanced or metastatic TNBC	ASCENT (NCT02574455)
Dec-2020	Margetuximab-cmkb (MARGENZA, MacroGenics)	Combined with chemotherapy	Metastatic HER2^+^ breast cancer	SOPHIA (NCT02492711)
June-2020	PHESGO (Genentech, Inc)	Neoadjuvant treatment	HER2^+^ breast cancer	FeDeriCa (NCT03493854)
April-2020	TUKYSA (Seattle Genetics, Inc)	Combined with trastuzumab and capecitabine	HER2^+^ breast cancer	HER2CLIMB trial (NCT02614794)
Feb-2020	Neratinib (NERLYNX, Puma Biotechnology, Inc)	Combined with capecitabine	HER2^+^ breast cancer	NALA (NCT01808573)
May-2019	PIQRAY^®^ (alpelisib)	Combined with fulvestrant	HR^+^, HER2^-^, PIK3CA-mutated advanced or metastatic breast cancer	SOLAR-1 (NCT02437318)
May-2019	Ado-trastuzumab emtansine (KADCYLA, Genentech, Inc)	Adjuvant treatment	HER2^+^ early breast cancer	KATHERINE (NCT01772472)
March-2019	Atezolizumab (TECENTRIQ, Genentech Inc)	Combined with paclitaxel protein-bound	Unresectable locally advanced or metastatic TNBC with PD-L1 expression	IMpassion130 (NCT02425891)
Oct-2018	Talazoparib (TALZENNA, Pfizer Inc)	——	Deleterious or suspected deleterious germline BRCA-mutated, HER2^-^ locally advanced or metastatic breast cancer	EMBRACA (NCT01945775)
Jan-2018	Olaparib tablets (Lynparza, AstraZeneca Pharmaceuticals LP)	——	Deleterious or suspected deleterious germline BRCA-mutated, HER2^-^ metastatic breast cancer	OlympiAD (NCT02000622)
Dec-2017	Pertuzumab (PERJETA, Genentech, Inc)	Combined with trastuzumab and chemotherapy as adjuvant treatment	HER2^+^ early breast cancer	APHINITY (NCT01358877)
Dec-2017	OGIVRI^®^ (trastuzumab-dkst)	——	HER2^+^ breast cancer	Comparisons of Herceptin
Sep-2017	Abemaciclib (VERZENIO, Eli Lilly and Company)	Combined with fulvestrant	HR^+^, HER2^-^ advanced or metastatic breast cancer	MONARCH 2
July-2017	Neratinib (NERLYNX, Puma Biotechnology, Inc)	Adjuvant treatment	Early stage HER2-overexpressed/amplified breast cancer	ExteNET trial (NCT00878709)

HR, hormone receptor; HER2, human epidermal growth-factor receptor 2; ER, estrogen receptor; TNBC, triple-negative breast cancer.

## 6 Conclusion

Overall, it can be stated that biomaterial-based immunotherapeutic strategies have shown great promise in breast cancer treatments, however, more investigations are needed to better design the biomaterials to achieve more precise treatment regimens for breast cancer patients possessing unique characteristics.

## Glossary

TNBC: triple negative breast cancer
ER: estrogen receptor
PR: progesterone receptor
HER2: human epidermal growth factor receptor 2
PD-L1: programmed death-ligand 1
TIL: tumor-infiltrating lymphocyte
CAR: chimeric antigen receptor
TCR: T-cell receptor
PD-1: programmed cell death protein 1
CTLA-4: cytotoxic T-lymphocyte-associated protein 4
APCs: antigen-presenting cells
FDA: U.S. Food and Drug Administration
TME: tumor micro-environment
CAFs: cancer-associated fibroblasts
ECM: extracellular matrix
NPs: nanoparticles
EPR: enhanced permeability and retention
Redox: oxidation-reduction
TAMs: tumor-associated macrophages
Tregs: regulatory T cells
MDSCs: myeloid-derived suppressor cells
MCSF: macrophage colony stimulating factor
CSF1-R: colony stimulating factor 1 receptor
SH2: Src homology region 2
DSPE-PEG: 1, 2-distearoyl-sn-glycero-3-phosphoethanolamine-poly(ethylene-glycol)
SIRPα: signal regulatory protein α
MAPK: mitogen-activated protein kinase
STING: stimulators of interferon gene
cyclic GMP-AMP: cyclic guanosine monophosphate–adenosine monophosphate
MHC: major histocompatibility complex
M2NPs: M2-like TAM dual-targeting NPs
SR-B1: scavenger receptor B type 1
M2pep: M2 macrophage binding peptide
TGF-β: transforming growth factor beta
DOX: doxorubicin
MRD: melittin-RADA32-doxorubicin
PI3Kγ: phosphatidylinositol 3-kinase γ
PTX: paclitaxel
CS: chitosan
GO: graphene oxide
HGF: hepatocyte growth factor
EGF: epidermal growth factor
MMPs: matrix metalloproteinases
FAP: fibroblast-activation protein
FSP: fibroblast-specific protein 1
DDR2: discoidin domain-containing receptor 2
PDGFRα/β: platelet-derived growth factor receptors alpha and beta
EMT: epithelial-mesenchymal transition
scFv: FAP-specific single chain variable fragment
CAP: cleavable amphiphilic peptide
Iri: irinotecan
C16-N: peptide C16-GNNQQNYKD-OH
Dox-L: DOX-loaded liposomes
nanoPue: puerarin nano-emulsion
AEAA: aminoethyl anis amide
ROS: reactive oxygen species
IFN-γ: interferon-γ
NF-κB: nuclear factor-κB
NO: nitric oxide
HA: hyaluronic acid
COS-ATRA: chitosan oligosaccharide-all-*trans*-retinoic-acid
PMN: pre-metastatic niche
LMWH-ATRA: low molecular-weight-heparin-all-*trans*-retinoic-acid
αGC: α-galactosylceramide
VECs: vascular endothelial cells
IDO: Indoleamine 2,3-dioxygenase
PVA: poly(vinyl alcohol)
NG: nanogel
DTX: docetaxel
VEA: vinyl ether acrylate
ICD: immunogenic cell death
NFX: nifuroxazide
CLM: co-prodrug-loaded micelles
LPD: lipid-protamine-DNA
pDNA: plasmid DNA
DOTAP: 1,2-dioleoyl-3-trimethylammonium propane chloride salt
PEG: polyethylene glycol
NK: natural killer cells
DCs: Dendritic cells
cDCs: conventional DCs
pDCs: plasmacytoid DCs
MoDCs: monocyte-derived DCs
PLGA: Poly-lactide-co-glycolic acid
MR: mannose receptor
TAAs: tumor associated antigens
CLR: C-type lectin receptor
NBs: nanobubbles
XCL1: XC-chemokine ligand 1
FLT3L: FMS-related tyrosine kinase 3 ligand
Ace-DEX: acetylated dextran
MPs: microparticles
PAMPs: pathogen associated molecular patterns
DOPC: 1,2-dioleoyl-sn-glycero-3-phosphocholine
DPPC: 1,2-dipalmitoyl-sn-glycero-3-phosphocholine
mPEG2000-DSPE: methoxy-poly (ethylene glycol)-1,2-distearoyl-sn-glycero-3-phosphoethanolamine-N
cdGMP: Cyclic diguanylate monophosphate
MPLA: mono-phosphoryl lipid A
TIM3: T cell immunoglobulin and mucin domain-containing 3
LAG3: lymphocyte activation gene 3
VISTA: V domain Ig suppressor of T cell activation
CHI: chidamide
ICB: immune checkpoint blockade
BSA: bovine serum albumin
FA: folic acid
RT: radiation therapy
PTT: photothermal therapy
CL: chitosan lactate
SPION: superparamagnetic iron oxide
EGFR: epidermal growth factor receptor
